# The efficacy of DNA barcoding in the classification, genetic differentiation, and biodiversity assessment of benthic macroinvertebrates

**DOI:** 10.1002/ece3.7470

**Published:** 2021-04-04

**Authors:** Yihao Ge, Chengxing Xia, Jun Wang, Xiujie Zhang, Xufa Ma, Qiong Zhou

**Affiliations:** ^1^ Key Laboratory of Freshwater Animal Breeding Ministry of Agriculture and Rural Affair/Key Laboratory of Agricultural Animal Genetics, Breeding and Reproduction Ministry of Education College of Fisheries Huazhong Agricultural University Wuhan China; ^2^ Engineering Research Center of Green Development for Conventional Aquatic Biological Industry in the Yangtze River Economic Belt Ministry of Education Wuhan China; ^3^ The Key Laboratory of Aquatic Biodiversity and Conservation Institute of Hydrobiology Chinese Academy of Sciences Wuhan China

**Keywords:** benthic macroinvertebrates, biodiversity assessment, DNA barcoding, species identification

## Abstract

Macroinvertebrates have been recognized as key ecological indicators of aquatic environment and are the most commonly used approaches for water quality assessment. However, species identification of macroinvertebrates (especially of aquatic insects) proves to be very difficult due to the lack of taxonomic expertise in some regions and can become time‐consuming. In this study, we evaluated the feasibility of DNA barcoding for the classification of benthic macroinvertebrates and investigated the genetic differentiation in seven orders (Insecta: Ephemeroptera, Plecoptera, Trichoptera, Diptera, Hemiptera, Coleoptera, and Odonata) from four large transboundary rivers of northwest China and further explored its potential application to biodiversity assessment. A total of 1,144 COI sequences, belonging to 176 species, 112 genera, and 53 families were obtained and analyzed. The barcoding gap analysis showed that COI gene fragment yielded significant intra‐ and interspecific divergences and obvious barcoding gaps. NJ phylogenetic trees showed that all species group into monophyletic species clusters whether from the same population or not, except two species (*Polypedilum. laetum* and *Polypedilum. bullum*). The distance‐based (ABGD) and tree‐based (PTP and MPTP) methods were utilized for grouping specimens into Operational Taxonomic Units (OTUs) and delimiting species. The ABGD, PTP, and MPTP analysis were divided into 177 (*p* = .0599), 197, and 195 OTUs, respectively. The BIN analysis generated 186 different BINs. Overall, our study showed that DNA barcoding offers an effective framework for macroinvertebrate species identification and sheds new light on the biodiversity assessment of local macroinvertebrates. Also, the construction of DNA barcode reference library of benthic macroinvertebrates in Eurasian transboundary rivers provides a solid backup for bioassessment studies of freshwater habitats using modern high‐throughput technologies in the near future.

## Graphical abstract

We evaluated the feasibility of DNA barcoding for the classification of benthic macroinvertebrates from four large transboundary rivers of northwest China. Our study shows that DNA barcoding can be used to clarify species boundaries and improve the reliability of environmental and biodiversity assessment in rivers.

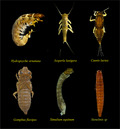

## 1 INTRODUCTION

Macroinvertebrates (e.g., aquatic insects) are considered as one of the primary components in aquatic ecosystems and represent a key group as food resources for higher trophic levels (Castella et al., 1984). They provide unique ecosystem services in nutrient cycling as well as energy flow (Sivaramakrishnan et al., 2014). Macroinvertebrates have been frequently utilized to monitor the water quality worldwide (Chandler, 1970; Helson & Willians, 2013), especially for those water bodies (e.g., rivers and lakes) affected by anthropogenic activities (Wang et al., 2006; Zhang et al., 2011). However, these monitoring functions depend, to a large extent, on the accuracy and precision of species/specimen identification (Frézal & Leblois, 2008; Macher et al., 2016). Traditional taxonomy relies on the morphology‐based traits to identify a species, but this approach proves to be difficult, time‐consuming, and costly (Wong et al., 2014). Although the need for species‐level identification in biomonitoring is controversial (Bailey et al., 2001; Lenat & Resh, 2001), DNA barcoding can provide the option of species‐level identification when taxonomic discrimination at the species level is warranted. Moreover, the increased taxonomic resolution delivered by DNA barcoding will provide more sensitive measures of the magnitudes and types of environmental impacts (Pfenninger et al., 2007). The morphological delineation for aquatic insects, in particular of females and immatures, remains a challenging task, as some important traits for reliable identification are only available in a single sex or at a certain stage of development (Zhou et al., 2009a; Zhou et al., 2011). For example, the morphological identification of the mosquito *Anopheles funestus* and its sibling species can only be distinguished at specific stages of their development (Cohuet et al., 2003). Unlike those model species such as butterflies (Dinca et al., 2015) and beetles (Kang et al., 2012), the information on aquatic insects is quite limited due to the great variety of species and complex life history (Morse et al., 1984). Despite the increasing demand for well‐trained taxonomists to support the assessment of aquatic ecosystems, the number of ecologists and researchers with taxonomic expertise is decreasing (Haase et al., 2006). Accordingly, such factors may hinder the accurate description of macroinvertebrates in terms of traditional classification.

To circumvent the morphological hurdles, DNA barcoding sensu Hebert et al. (2003) utilizes a short standard sequence of the mitochondrial genome for the species identification and classification, based on a part of the cytochrome c oxidase subunit I (COI) gene (Hebert, Cywinska, et al., 2003; Moriniere et al., 2017). DNA barcoding has the potential to identify cryptic species and highlight the diversity of macroinvertebrates in aquatic ecosystems (Bucklin et al., 2010), providing valuable information on the selection of taxa for further analyses (Hajibabaei et al., 2006) and allowing an identification of all life history stages and genders of a species (Casiraghi et al., 2010; Murria et al., 2010). Thus, DNA barcoding provides an effective way to overcome the difficulties in traditional morphological delineation, as this technology delivers fast, efficient, and reliable species identification (Kress et al., 2012). However, it is noted that various problems may affect the use of mitochondrial DNA, for example, Wolbachia infections (Werren et al., 1995), heteroplasmy (Kavar et al., 2006), introgressive hybridization (Raupach et al., 2014), incomplete lineage sorting (Petit & Excoffier, 2009), pseudogenes (Ribeiro Leite, 2012), and the recent speciation events (Raupach et al., 2014), and thus affect the efficiency of DNA barcodes to discriminate the analyzed species (Havemann et al., 2018). Unknown specimens could be identified if their DNA barcode sequences match in comparison with a given barcode reference library (Moriniere et al., 2017). Previous studies illuminate the great reliability of DNA barcoding in species identification of aquatic insects, for example, Ephemeroptera (Ball et al., 2005; Curt et al., 2012), Plecoptera and Trichoptera (Gill et al., 2014), Hemiptera (Havemann et al., 2018) and Diptera (Brodin et al., 2012; Hernandez‐Triana et al., 2014; Hunter et al., 2008; Lin et al., 2015). However, these studies focus primarily on the application of DNA barcoding in one to three orders, and few studies consider all the taxa of macroinvertebrates in a specific ecosystem and further apply to the environmental and biodiversity assessment (Ball et al., 2005; Moriniere et al., 2017).

Aquatic insects are predominant faunas in the transboundary rivers of northwest China (Wang et al., 2014). Despite extraordinarily abundant resources for aquatic insects, few studies have been carried out in these transboundary rivers (Wang et al., 2014). China exhibits highly different characteristics of climate and geography that harbors an enormous diversity in aquatic biota. Thus, great variability in species distribution and high diversity in aquatic biota occur in China. Until now, DNA barcoding has been applied to some specific taxa, for example, amphibians (Che et al., 2012), birds (Yoo et al., 2013), plants (Huang & Ke, 2015), Noctuoidea moths (Yang et al., 2014), mollusks (Barco et al., 2016), crickets (Hawlitschek et al., 2017), herpetofauna (Hawlitschek et al., 2017), fish (Smith et al., 2008), spiders (Ivanov et al., 2018), but rarely focuses on aquatic invertebrates (Zhou, 2009b; Zhou et al., 2009a). In this study, we attempted to test the feasibility of DNA barcoding in the classification of benthic macroinvertebrates in the transboundary rivers and their affiliated water bodies of northwest China. The main objectives of this study were to establish a reliable DNA barcode reference library for benthic macroinvertebrates in Eurasian transboundary rivers and sheds new light on the diversity status of local macroinvertebrates.

## 2 MATERIALS AND METHODS

### 2.1 Study area

**FIGURE 1:**
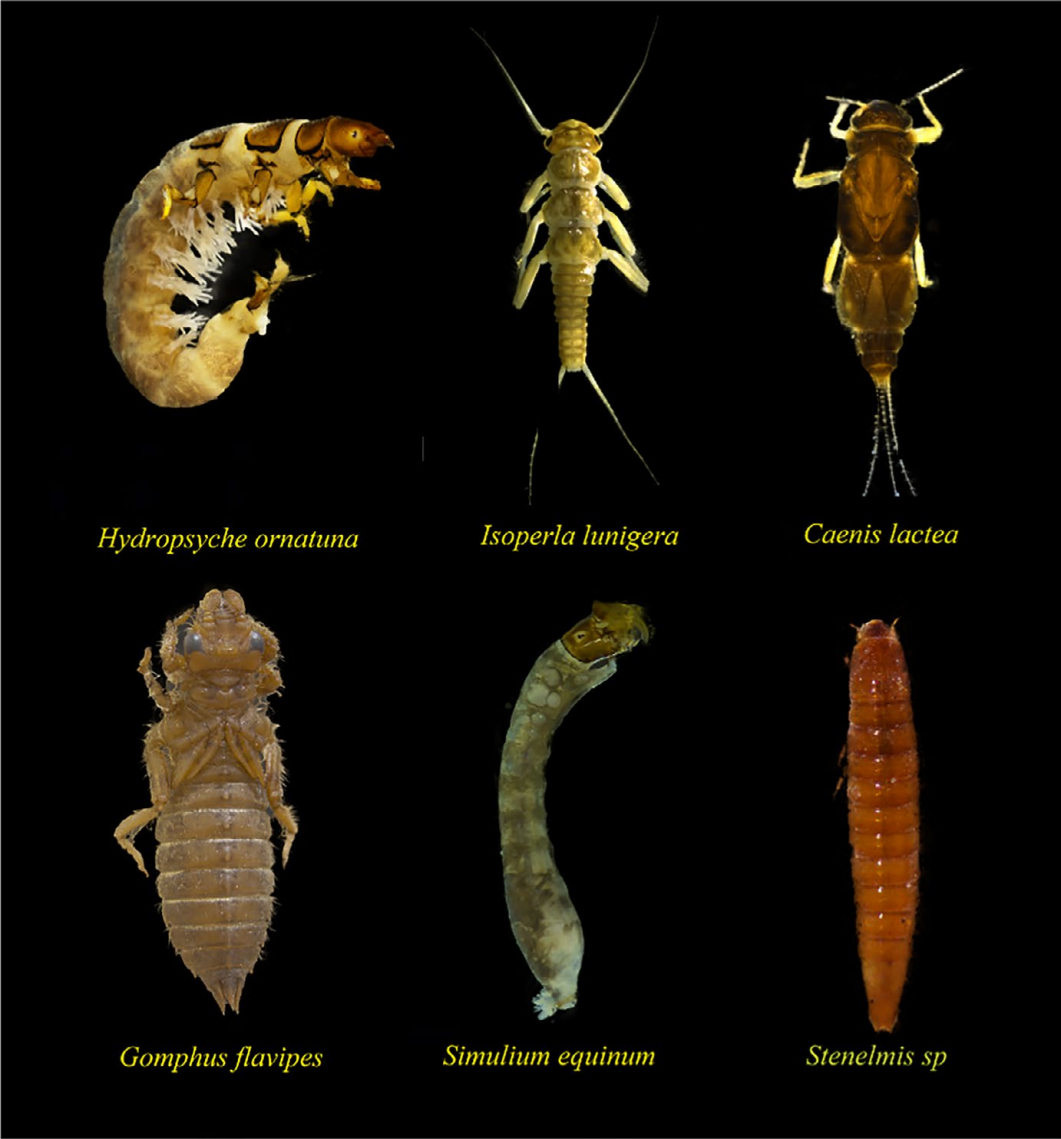
Selected common species of benthic macroinvertebrates in four transboundary rivers of northwest China

**FIGURE 2:**
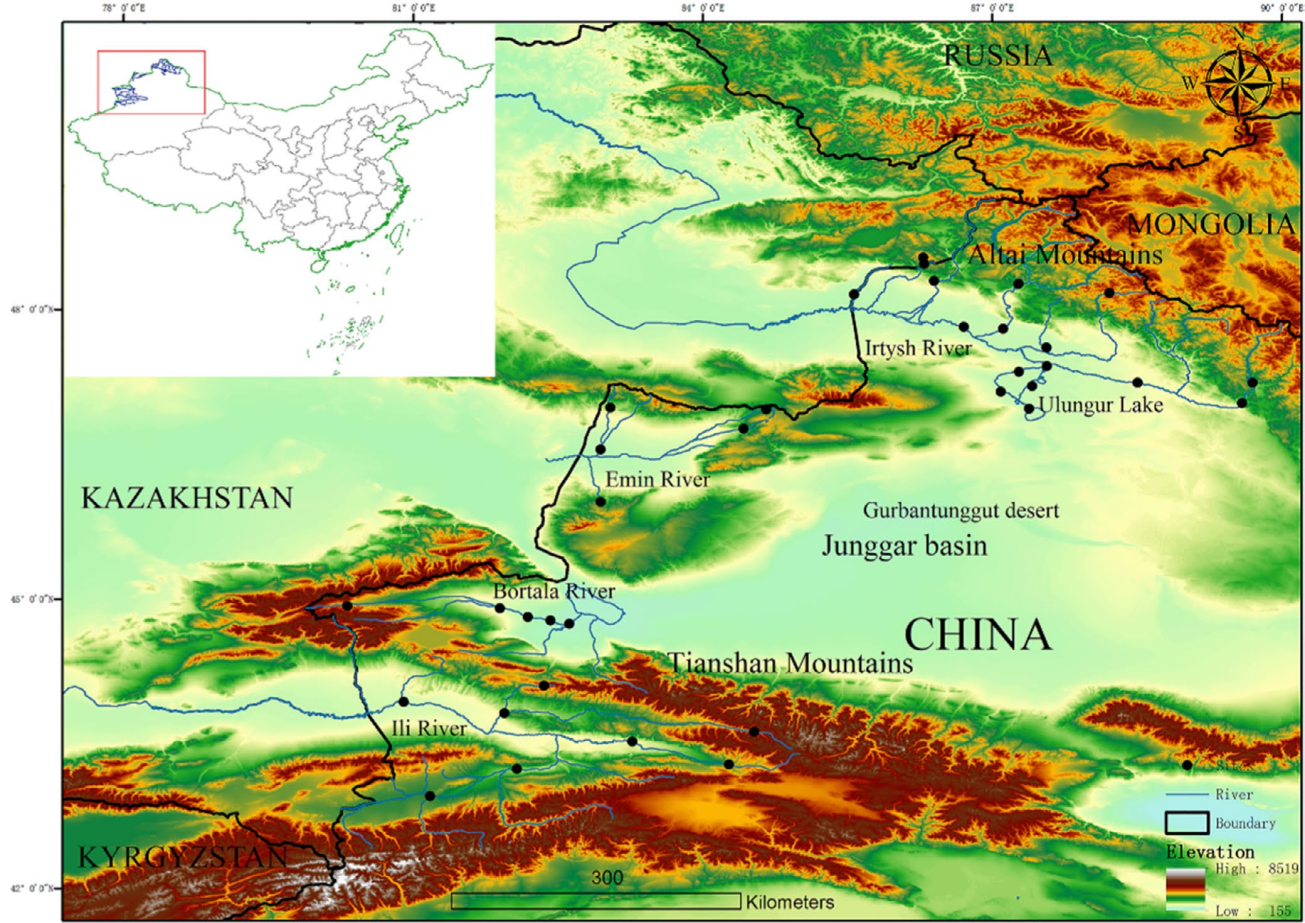
Sketch map showing the sampling locations of benthic macroinvertebrates in four transboundary rivers of northwest China. Solid black circles and solid black line represent the sampling sites of benthic macroinvertebrates and the border among different countries, respectively. The blue line in the middle of the map represents the rivers and main branches (Irtysh River, Emin River, Bortala River, and Ili River)

Our study was conducted in Xinjiang Uygur Autonomous Region (Figure 1), center of Eurasia (Figure 2). This region extends from the south slope of the Altai Mountains to the hinterland of the Tianshan Mountains. The Junggar basin between the two mountains is covered by the Gurbantunggut desert, the second largest desert in China. This region belongs to semi‐arid and arid climatic zone, with the elevation ranging from 189 to 7,435 m, and encompasses complex landforms and microclimate between mountains and basins. Due to the characteristics of geography, environment, and climate, Xinjiang region provides biological communities with a variety of habitats and has been listed as one of the priority areas for biodiversity conservation in China. The Irtysh River, Emin River, Ili River, and Bortala River originate from Xinjiang Uygur Autonomous Region, northwest China (Figure 2) and ultimately flow toward the Republic of Kazakhstan. In particular, the Ili River is the largest river in Xinjiang region. The Irtysh River is the only Chinese river that discharges into the Arctic Ocean. Ulungur Lake covers an area of 1,035 square kilometers (Wang, 2010).

### 2.2 Sampling and experimental material

We collected macroinvertebrate samples in four transboundary rivers (the Irtysh River, Emin River, Ili River, and Bortala River) between China and Kazakhstan, Mongolia and Russia (Figure 2). Study sites were set in the main stems, tributaries and affiliated water bodies (lakes and reservoirs). Due to a long‐frozen winter season (November to April), mayflies (Ephemeroptera), stoneflies (Plecoptera), caddisflies (Trichoptera), true flies (Diptera), true bugs (Hemiptera), beetles (Coleoptera), and dragonflies (Odonata) were collected in May, July, August, and October of 2013–2017. Macroinvertebrates were collected by Surber net, Kick‐net, Peterson grab, and D‐framed dip net according to the habitat type of study areas. Standardized sampling protocols were executed at different habitats (i.e., lentic and lotic water bodies) from upstream to downstream (Stark et al., 2001). Approximately 30,000 specimens were preserved in 95% ethanol to allow for the morphological identification and molecular analyses. In some cases, ethanol was replaced for two or three times in order to guarantee a good preservation of the specimens that can be used for further molecular analysis. Macroinvertebrate samples were sorted and identified under a dissecting microscope in the laboratory, and stored at −20°C at the College of Fisheries, Huazhong Agricultural University (Brinkhurst, 1986; Dudgeon, 1999; Epler & Epler, 2001; Morse et al., 1994; Zhou et al., 2003). In the laboratory, specimens were identified based on morphological characteristics. Intact individuals were selected to conduct the DNA barcoding analysis.

### 2.3 DNA extractions, amplification and sequencing

Total genomic DNA was extracted from legs, cerci, half or whole bodies according to a specimen's size, using the phenol–chloroform protocol (Barnett & Larson, 2012) and quantified in a NanoDrop 2000c (Thermo Fisher Scientific, USA). The primer pair LCO‐1490 (5’‐GGTCAACAAATCATAAAGATATTGG‐3’) and HCO‐2198 (5’‐TAAACTTCAGGGTGACCAAAAAATCA‐3’) (Folmer et al., 1994) was used to amplify the DNA barcode fragment with the length of about 658 base pairs (bp). Polymerase Chain Reactions were performed in 50 µl volumes using the following reagents and concentrations: containing 10 × PCR buffer 5 μl, Mg^2+^ (2.5 mmol/L) 5 μL, dNTP (2.5 mmol/L) 3.5 μl, l.5 μl (10 nmol/L) each primer, 0.5 U Taq polymerase (Takara, Dalian, China), 2 μl (50 ng/μl) DNA template and complete ddH_2_O to 50 μl. The PCRs were run as follows: 94°C for 3 min; then 94°C for 1 min, 45°C for 2 min, and 72°C for 3 min, for 40 cycles; and 72°C for another 5 min (Folmer et al., 1994). All PCR products were checked by electrophoresis at 1% agarose with an ethidium bromide stain, and if present, the PCR products were subsequently purified by ExoI/FastAP (Fermentas) and directly sequenced by Invitrogen Corporation in China.

### 2.4 Barcoding analysis based on COI

Bidirectional sequencing was employed to maximize the precision of sequencing. We aligned the COI gene using the ClustalW program from MEGA X (Kumar et al., 2018) package for each order with default parameters (Zhou et al., 2010). The online version of MAFFT v. 7.0 (Katoh & Standley, 2013) was utilized to align COI gene sequences under the algorithm Q–INS–I and the rest set as default. Amino acid translation was conducted to ensure that no gaps or stop codons existed in the alignment. We obtained 1,144 COI sequences from 176 species in total. Detailed information (locality data, habitat, altitude, collector, identifier, taxonomic classifications, habitus images, and DNA barcode sequences) for each voucher specimen was deposited to the Barcode of Life Data System at http://www.boldsystems.org (Ball et al., 2005), under the dataset project “XJDQD” (Process IDs: XJDQD001‐18‐XJDQD1275‐18).

#### 2.4.1 Phylogenetic tree

Maximum likelihood (ML) analyses in MEGA 10.0 software was used for tree construction. When the multiple sequence alignment with ClustalW was complete, we used the “models” function to determine the best DNA/Protein Models. A total of 1,000 nonparametric bootstrap replicates were used (Felsenstein, 1985). To evaluate the accuracy of DNA barcoding for the classification and evolutionary relationship of species, we downloaded the sequencing data of relevant species from BOLD (Raupach et al., 2014; Stein et al., 2014) (Part I, Supplementary Material). A total of 1,000 replicates were utilized to detect the reliability of each branch of the tree in order to obtain bootstrap values.

#### 2.4.2 Barcode Gap Analysis and BIN analysis

Genetic distances within species and genera, determined using the Kimura 2‐parameter (K2P) (Kimura, 1980) distance, were inputted into the MEGA X program (Kumar et al., 2018). Barcode Gap Analysis in the BOLD system was carried out to compare the distribution of distances within each species and the distance to the nearest neighbor of each species. Species were tested for the presence of barcode gaps between maximum intraspecific genetic distance and minimum interspecific distance according to Ratnasingham and Hebert (2007). Within the BOLD, the BIN system groups sequence data into clusters of closely COI barcode sequences that are assigned to a globally unique identifier, termed Barcode Index Number (BIN, Ratnasingham & Hebert, 2007). BIN analysis was restricted to sequences with a minimum length of 500 bp. Members of a BIN usually belong to a single species recognized by traditional taxonomy (Hendrich et al., 2015). Every case of disagreement/conflict is the starting point for reevaluation of both molecular and morphological data. We follow the concept of an integrative taxonomic approach to infer whether there are previously overlooked species in the samples or whether barcode divergences between species are large enough to enable delineation of species using the usual partial COI sequence (Moriniere et al., 2017). BINs for sequence clusters enable the delineation of geographical clades, which might reflect local environmental features (Moriniere et al., 2017).

#### 2.4.3 Automatic Barcode Gap Discovery analysis

Automatic Barcode Gap Discovery (ABGD) analysis (Puillandre et al., 2012) was used to group specimens into operational taxonomic units (OTUs) based on DNA barcodes and applied to compare the results of the BIN‐based Barcode Gap Analysis, which was run by a web interface (www.abi.snv.jussieu.fr/public/abgd/). The default value for the relative gap‐width was set as x = 1.5. Moreover, we analyzed each order in a separate analysis. All the assignments that the values (P) of intraspecific divergence ranged between 0.001 and 0.1 were recorded. Default settings were employed for all remaining parameters.

#### 2.4.4 Analyses of Poisson Tree Processes (PTP) and Multiple‐threshold PTP (MPTP)

Phylogenetic inference analyses were conducted using maximum likelihood optimality criteria. ML analyses were conducted using RAXML 8.2.9 (Stamatakis, 2014) with 1,000 nonparametric bootstrap replicates. Appropriate substitution models were determined by JMODELTEST 2 (Darriba et al., 2012), and the best fitting substitution models were GTR + I + G. Poisson tree process (Zhang et al., 2013) was used in species delimitation analysis. Since PTPs belong to tree‐based methods, they require species monophyly and are based on the analysis of branching rates (Fontaneto et al., 2015). Single‐threshold Bayesian PTP analyses (Zhang et al., 2013) were conducted on the website http://species.h‐its.org/ptp. The analyses were run for 1,000,000 MCMC generations with thinning value = 100 and burn‐in = 0.25. The trace files were checked for the convergence of the MCMC. Multiple‐threshold PTP (MPTP) analyses (Kapli et al., 2017) were run on the website https://mptp.h‐its.org. Support information for the species delimitation hypotheses obtained with the ML implementation of PTP are shown in Appendix S5.

## 3 RESULTS

### 3.1 Sequencing analyses

A total of 1,678 individuals were collected and utilized for the generation of DNA barcodes. However, 451 specimens showed poor quality and were excluded from further analyses. Accordingly, a total of 1,144 sequences (Ephemeroptera (*n* = 516), Plecoptera (146), Trichoptera (81), Hemiptera (66), Diptera (273), Coleoptera (55), and Odonata (23)), belonging to 176 species, 112 genera, and 53 families, were successfully generated. The lengths of all COI sequences were no less than 600 bp for sequencing analysis. Deletions, insertions, and stop codons were not detected in the sequences. All the species exhibited high adenosine and thymine (AT)‐rich bias (averaged 63.1%) as it is typically known from arthropods. Specifically, the A + T content in Trichoptera was up to 69.9% and the lowest was shown in Diptera (55.6%).

### 3.2 Genetic distance

**FIGURE 3:**
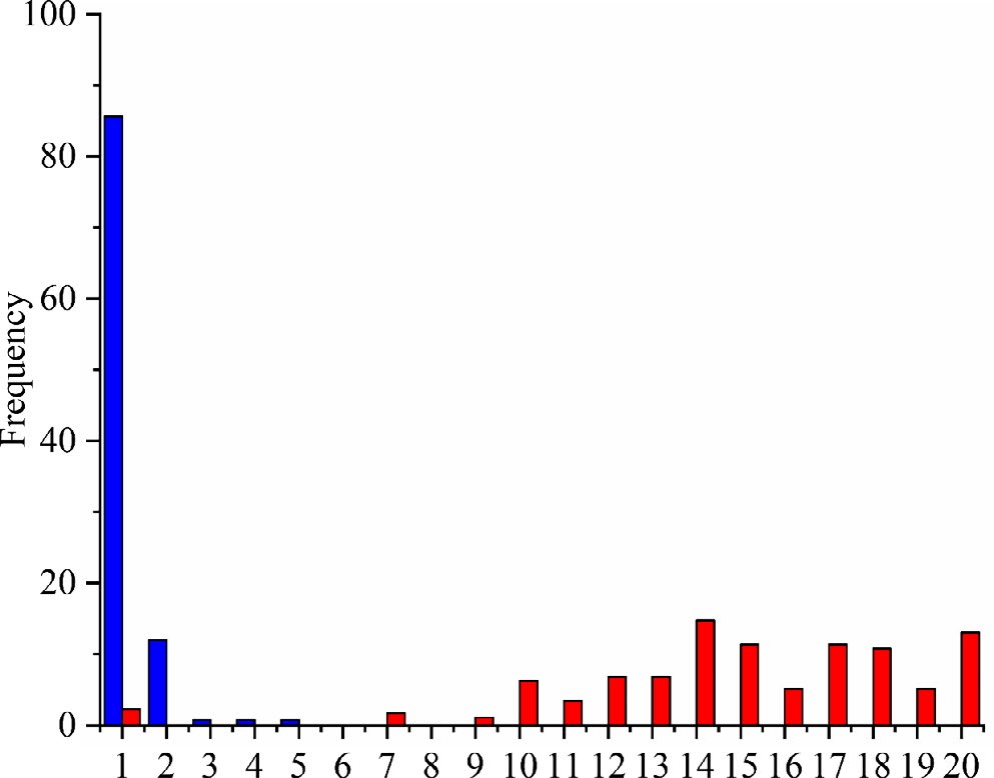
The distribution histograms of mean intraspecific distance (in blue) and the distance to nearest neighbor (in red) based on Kimura 2‐parameter distance

**FIGURE 4:**
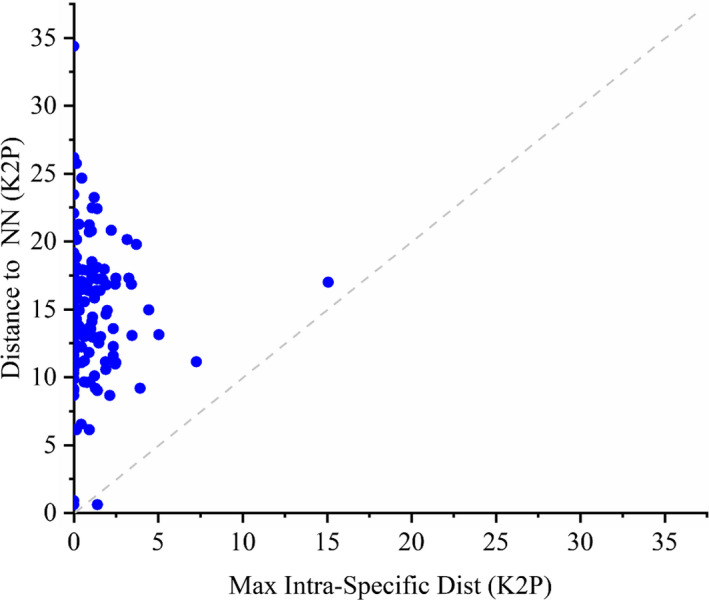
Barcode gap plot showing the distance to the nearest neighbor (NN) versus. the maximum intraspecific distance Kimura 2‐parameter (K2P) for 189 species. Dots above the 1:1 line indicate the presence of a barcode gap

The level of genetic divergence in the COI genes is summarized in Table S1. Intraspecific K2P distances ranged from 0% to 15.1% with an average of 0.78%, whereas interspecific K2P distance within a genus ranged from 0.6% to 33.4% (average 16.4%). Interspecific K2P distance within one genus was 21‐fold higher than those of intraspecific K2P distances. Although the maximum intraspecific distance and the distance to the nearest neighbor overlapped partially (Figure 3), the averages of the nearest neighbor distances were 28‐fold higher than those of the maximum intraspecific distances. Based on the Barcode Gap analysis, the minimum interspecific distance for 174 species was larger than the maximum intraspecific distance (98.9%; Figure 4). For the species *Polypedilum. laetum* and *Polypedilum*. *bullum*, the maximum intraspecific variations overlapped with the NN distance, leading to the absence of a barcode gap. For the species *Chironomus heterodentatus* and *Chironomus* sp. XJ, the distances to the NN were less than 2% of sequence divergence, but more than the maximum intraspecific value. We compared the means of intraspecific K2P distance for seven macroinvertebrate taxonomic groups (Table S1). Mollusks exhibited the largest mean intraspecific distance (0.89%), whereas the analyzed Odonata yielded the lowest value of 0.28%.

### 3.3 Phylogenetic tree‐based identification and cluster analysis

**FIGURE 5:**
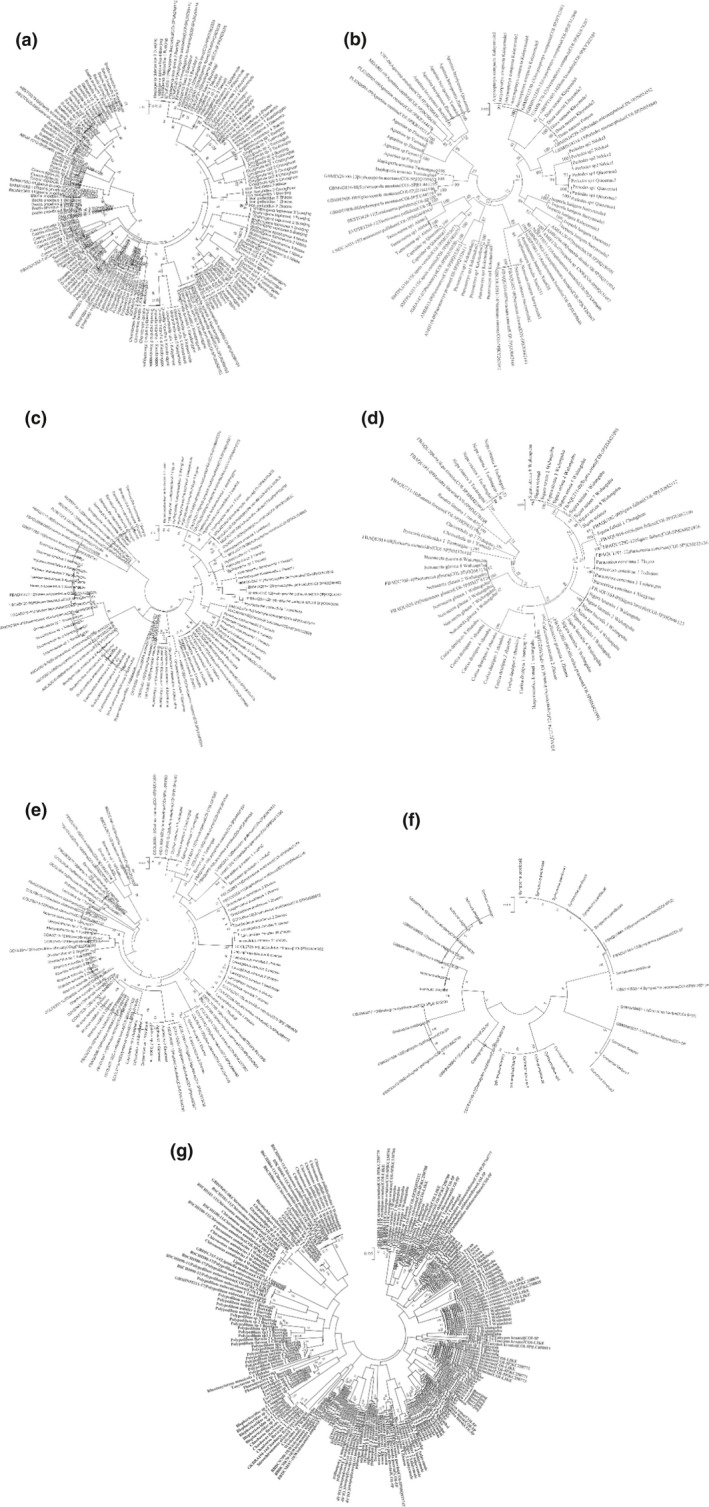
The maximum likelihood trees of Ephemeroptera (a), Plecoptera (b), Trichoptera (c), Hemiptera (d), Coleoptera (e), Odonata (f), and Diptera (g) based on K2P distance

Through the ML method, phylogenetic trees of seven orders show that all individuals grouped into monophyletic species clusters with high bootstrap values expect *P. laetum* and *P. bullum* (Figure 5). For those species with two or more representatives, the conspecifics in monophyletic clades were associated with high confidence (100% bootstrap in the NJ tree). Although the very low interspecific variations (0.62%) were observed, species *Chironomus* sp. XJ and *Chironomus heterodentatus* could be well distinguished based on phylogenetic analyses. An unusual cluster was revealed for *P. bullum*, where it clustered with five individuals of *P. laetum* with a 0.63% mean interspecific distance (ranged 0.21%‐1.06%). Species *Dicranota guerini*, *Ameletus montanus, Glyptotendipes* sp. XJ*, Euryhapsis* sp*, Cricotopus ornatus, Atherix* sp. XJ, and *Epeorus* sp5 exhibited large internal splits in their monophyletic clusters (with > 90% support values), and large K2P distances were detected between the internal clusters (Figure S1). In addition, the clades of *Sigara striata* and *Chironomus pallidivittatus* were formed with some subclusters with relatively large mean K2P genetic distance (Figure S2).

The clustering analysis of 265 individual sequences from the BOLD data show that the species were clustered into species‐specific groups with the homologous specimens (Figures 5a–5g). Firstly, the specimens of the same populations in our study were clustered together, and then were clustered with those from other areas (Germany, United States, Mexico, Canada, Norway, Italy, Finland, Japan, Iran etc). Based on the BLAST results, the query coverage and identity of COI sequences for the species in this study compared with those submitted in BOLD database did not reach 100%.

### 3.4 OTU delineation based on distance using ABGD

ABGD software was utilized to delineate 176 morphological species and in total, 1,144 sequences with the assignments for intraspecific divergence (p) values between 0.001 and 0.1. The results included two cases: initial partition and recursive partition. As revealed in Part II (Supplementary Material), 1,144 samples were divided into 216 groups (*p* value: .0028–.0077) and 177 groups (*p* value: .0129–.0599) in two cases, respectively. The ABGD analysis derived a total of 177 OTUs with a prior intraspecific divergence of *P*_max_ = 0.0599. Therefore, we selected the initial results to compare with those of morphological identification. We found that *A. montanus* and *D. guerini* were grouped into two different groups, whereas *Chironomus s*p. XJ and *C. heterodentatus*, *P. bullum* and *P. laetum*, *Diamesa* sp. XJC and *Diamesa* sp. XJB were clustered into the same group.

### 3.5 Tree‐based (PTP and MPTP) delimitation analysis

The groups delimited by the PTP and MPTP analysis was much more than that of recognized morphospecies. The PTP and MPTP analyses, as the implementation of ML, generated 197 and 195 MOTUs, respectively (Supplementary Material Part III). Most incongruences between the delimited groups and morphology‐based identifications could ascribe to multiple molecular lineages/clusters. These incongruence cases were generally associated with high intraspecific genetic distance and were mainly reported in ten taxa (*Epeorus sp5, Rhithrogena tianshanica, Ameletus montanus, Atherix sp. XJ, Glyptotendipes sp. XJ, Dicranota guerini, Caenis lactea, Ischnura elegans, and Tabanus cordiger*).

### 3.6 BIN analysis

The 1,144 records generated a total of 186 different BINs. In particular, 117 BINs were not recorded previously in the BOLD database, and most sequences representing new endemic species had new BIN assignments. The Diptera species *Chironomus* sp. XJ and *C. heterodentatus* shared the same BIN [AAW4009], and *P. bullum* shared a BIN assignment [ACB4789] with *P. laetum*. BIN Discordance analysis was performed on BOLD (February 2018), and the results showed that two BINs were discordant with our prior taxonomic assignments, which indicated shared haplotypes and a low interspecific divergence. A number of 129 BIN clusters were found to be taxonomically concordant with other barcoding data on BOLD, and they were assigned to the same species. A number of 56 records are singletons, implying that these BINs were represented by only one sequence. Eight species were assigned to a total of 19 BINs (Table S2).

## 4 DISCUSSION

The species identification using DNA barcoding is based on the principle that the genetic distance between two species is much greater than that within a species. It has been proposed that 2% is the threshold value of species delimitation and, in general, the average genetic distance between two species is over 10 times of that within a species (Hebert, Cywinska, et al., 2003; Ward, 2009). In this study, the average of interspecific K2P distance (16.37%) was 21‐fold higher than that of intraspecific K2P distance (0.78%), which meets the criteria that the average of interspecific genetic distance is 10 more times than that of intraspecific genetic distance. The distribution histogram of intraspecific and interspecific distances shows that 85.19% and 97.04% of the intraspecific distances were less than 1% and 2%, respectively, and 97.88% of the interspecific distances were greater than 6%, implying a very little overlap between intraspecific and interspecific genetic distances. Based on the Barcode Gap analysis, the minimum interspecific distances to the nearest neighbor were larger than the maximum intraspecific distance for 174 species (98.9% of all species). Only for two species (*P. laetum* and *P. bullum*), the maximum intraspecific distances overlapped with the NN distance, leading to the absence of a barcode gap. The multi‐approach species delimitation showed a relatively high congruence between molecular groups and the Linnaean taxa with COI DNA barcodes. These results reveal that DNA barcoding based on COI gene is an effective method for the species identification of benthic macroinvertebrates in the transboundary rivers of northwest China.

DNA barcoding has been considered to be a successful molecular identification tool for insects such as mosquitoes (Cywinska, Hunter, & Hebert, 2006; Kumar et al., 2018; Weigand et al., 2019). Also, our study shows that DNA barcoding can offer a reliable framework for the identification of mosquito species even though the identifications of a few closely related species remain ambiguous (Versteirt et al., 2015). In this study, with the two closely related mosquito species (*Polypedilum*. *laetum* and *Polypedilum. bullum*), the maximum intraspecific distances overlapped with the NN distance, leading to the absence of a barcode gap. The absence of barcoding gap is not uncommon in mosquitoes (Cywinska, Hunter, & Hebert, 2006; Versteirt et al., 2015; Lin et al., 2015). These two mosquito species belong to the subgenus *Polypedilum* (Kieffer, 1912) and have overlapped distribution in western China. Based on the description of morphological characteristics, *P. laetum* (Meigen, 1804) should be the sister species of *P. bullum* (Zhang & Wang, 2004). The DNA barcode data show that recent speciation events as well as hybridization may represent important processes between *P. laetum* and *P. bullum*. Further in‐depth studies including more specimens and other genetic markers should be investigated to resolve the eco‐evolutionary events leading to the low interspecific variation.

Based on the NJ tree, ABGD, PTP, MPTP, and BIN analysis, high levels of genetic distance and multiple lineages were observed in eight taxa (*Epeorus* sp5*, Rhithrogena tianshanica, Ameletus montanus, Atherix* sp. XJ*, Glyptotendipes* sp. XJ*, Euryhapsis* sp*, Dicranota guerini and Cricotopus ornatus*), suggesting the presence of cryptic species of benthic macroinvertebrates in these transboundary rivers of northwest China. Although Hebert and Ward proposed the threshold value (2%) of species differentiation based on DNA barcoding (Hebert, Cywinska, et al., 2003; Ward, 2009), the differences in genetic differentiation can occur in different geographical populations for the same species, and thus, the genetic distance can exceed the threshold value of 2% for species classification (Hickerson et al., 2006; Tajima, 1989; Ward, 2009). In the present study, the eight species exhibited high intraspecific genetic distance and multiple genetic lineages, which was consistent with the conclusions in Ward (2009). Meanwhile, our results supported the conclusion that the genetic distance between different geographical populations of the conspecifics can exceed 2% (Hebert Ratnasingham & deWaard, 2003; Hebert, Cywinska, et al., 2003; Ward et al., 2005). Coincidentally, the two or three respective molecular lineages/clusters observed in *Glyptotendipes* sp. XJ, *C. ornatus*, *D. guerini*, *Atherix* sp. XJ, *R. tianshanica*, *Epeorus* sp5 and *A. montanus* corresponded to different geographical areas, implying that biogeographic events may result in a great intraspecific divergence for these species. Moreover, geographical isolation plays an important role in the formation of high intraspecific genetic distance or cryptic species.

We observed high intraspecific genetic distance (up to 15.07%) for species *Ameletus montanus*. Statistical methods (ABGD, BIN, PTP, and MPTP) divided them into two different OTUs. The presence of two clusters or OTUs with deep divergences that were observed in mayfly species was indicative of cryptic diversity. Actually, cryptic diversity is typical in mayfly species (Suh et al., 2019). High genetic divergences within nominal species can be interpreted as misidentification and, more importantly, as cryptic or unrecognized speciation events (Chen et al., 2015). Recognizing cryptic diversity contributes to improving our knowledge regarding the biodiversity of numerous taxa (e.g., benthic macroinvertebrates). The genetic differentiation within one species occurred at different sample sites or geographic scales for the Irtysh river, Emin river, and Ili river. For instance, the species *D. guerini* showed high intraspecific divergence (up to 7.26%) between the Irtysh River and Ili River populations. These findings probably suggest that geographical isolation and diversification events allow different populations to evolve in different directions, thus lead to a great increase in the diversity of benthic macroinvertebrates. This suggests that DNA barcodes could be supplemented in population genetics research, with morphological, ecological, nuclear DNA, and other nonmolecular data to explore the presence of cryptic species and assess intraspecific differentiation.

DNA barcoding has been widely used for species identification (Barco et al., 2016; Versteirt et al., 2015). However, whether DNA barcoding can distinguish the individuals from different geographical populations, subspecies or biotype, remains unknown. In this study, the NJ tree shows that the conspecifics of barcoding sequences firstly clustered together, and then clustered with those of other areas (Germany, United States, Mexico, Canada, Norway, Italy, Finland etc.). In the NJ tree, both *Sigara striata* and *Chironomous pallidivittatus* covered two subclusters, and this is in accordance with the sampling locations. The same geographical populations clustered together with high support values. It indicated that the evolution of geographical population was related to geographical distance. As a result, we inferred that the population differentiation of benthic macroinvertebrates in these four rivers was ascribed to geographical isolation. It has been reported that COI genes are not sensitive enough to identify intraspecific variation, especially when the geographical differentiation of populations is not high enough to form a single pattern (Aliabadian et al., 2009; Verheyen et al., 2003). This phenomenon was observed in our study. The genetic structure analysis among different geographic population show that the shared haplotypes existed in three adjacent geographic population of three mayfly species, whereas different geographical populations had a certain degree of gene flow, intrapopulation, and interpopulation genetic divergence. In the NJ tree, the geographical populations were not divided into different branches following geographical locations. The low level of genetic differentiation among populations decreased the reliability of COI gene to effectively distinguish intraspecific category. In contrast, with the accumulation of genetic differentiation among populations, DNA barcoding could be used to distinguish the geographical populations, subspecies or biotype (Monaghan et al., 2006). Although COI gene has great potential of species identification at a species level, but for infraspecific identification, the evolution rates of COI genes is limited because it is a protein‐coding gene. Therefore, COI gene is not sensitive enough to identify populations with tiny genetic differentiation, in which the geographical locations are adjacent and the formation of geographical isolation pattern is not long enough. In this case, more factors (e.g., increasing the length of DNA barcoding) could be considered, especially for those non‐protein‐coding genes with faster evolution rate.

This study is the first time to report the comprehensive DNA barcode reference library of benthic macroinvertebrates in Eurasian transboundary rivers. This library included nearly all of the dominant species appeared in environmental assessment studies (Figure 5). High‐throughput sequencing (HTS) can provide taxonomic information at greater resolution, depth, and consistency, and at lower cost than morphologically identified samples (Gibson et al., 2015). However, the application of HTS for taxonomic identification of samples in a biomonitoring context is limited by the availability of cytochrome c oxidase subunit 1 (COI) sequence records in reference libraries databases. Numerous investigators have reported that, without adequate representation in a reference library, accurate taxonomic identification for a given sequence can be very difficult (Ekrem et al., 2007). Therefore, our study enables future applications such as environmental DNA barcoding (Baird & Hajibabaei, 2012) and metabarcoding (Gibson et al., 2014) based on HTS and provides a solid backup for effective bioassessment in river and stream ecosystems.

## 5 CONCLUSION

In conclusion, our study revealed that DNA barcoding based on COI gene is an effective method to clarify species boundaries and quantitatively evaluate species diversity (e.g., taxa abundance and cryptic species). Population differentiation of benthic macroinvertebrates in four transboundary rivers was ascribed to geographical isolation. Geographical isolation and diversification events are two main factors for different populations to evolve in different directions and thus lead to a great increase in the diversity of benthic macroinvertebrates. Even so, DNA barcoding could be supplemented in population genetics studies, with morphological, ecological nuclear DNA, and other nonmolecular data regarding the existence of cryptic species and assessment of intraspecific divergence.

## CONFLICT OF INTEREST

The authors declare that they have no competing interests.

## AUTHOR CONTRIBUTIONS

**Yihao Ge:** Investigation (equal); Writing‐original draft (equal). **Chengxing Xia:** Investigation (equal); Writing‐original draft (equal). **Jun Wang:** Investigation (supporting); Methodology (supporting). **Xiujie Zhang:** Data curation (supporting); Formal analysis (lead); Methodology (supporting). **Xufa Ma:** Investigation (supporting); Methodology (supporting); Resources (supporting). **Qiong Zhou:** Conceptualization (equal); Data curation (lead); Funding acquisition (lead); Project administration (lead); Supervision (lead); Writing‐review & editing (lead).

## Supporting information

Supplementary Material

## Data Availability

Data used in this manuscript are catalogued in the Dryad Digital Repository (available at https://orcid.org/0000‐0001‐6293‐2928).
