# Supplementary material for: The efficacy of DNA barcoding in the classification, genetic differentiation, and biodiversity assessment of benthic macroinvertebrates
Source: Ecol Evol. 2021 Apr 4;11(10):5669–81. doi: 10.1002/ece3.7470 (PMC8131818; doi:10.1002/ece3.7470)

**Supporting Information**

For the research article:

**The efficacy of DNA barcoding in the classification, genetic differentiation and biodiversity assessment of benthic macroinvertebrates**

Yihao Ge^1,2,3#^, Chengxing Xia^1,2#^, Jun Wang^1,3^, Xiujie Zhang^1,2^, Xufa Ma^1,2^, Qiong Zhou^1,2^*****

1 College of Fisheries, Huazhong Agricultural University, **Key Laboratory of Freshwater Animal Breeding, Ministry of Agriculture,** Wuhan 430070, China; [geyihao@ihb.ac.cn](mailto:geyihao@ihb.ac.cn); [xiachengxing@mail.hzau.edu.cn](mailto:xiachengxing@mail.hzau.edu.cn); [zhangxj@mail.hzau.edu.cn](mailto:zhangxj@mail.hzau.edu.cn); [xufama@mail.hzau.edu.cn](mailto:xufama@mail.hzau.edu.cn); [hainan@mail.hzau.edu.cn](mailto:hainan@mail.hzau.edu.cn);

2 Hubei Provincial Engineering Laboratory for Pond Aquaculture, Wuhan 430070, China; [geyihao@ihb.ac.cn](mailto:geyihao@ihb.ac.cn); [xiachengxing@mail.hzau.edu.cn](mailto:xiachengxing@mail.hzau.edu.cn); [zhangxj@mail.hzau.edu.cn](mailto:zhangxj@mail.hzau.edu.cn); [xufama@mail.hzau.edu.cn](mailto:xufama@mail.hzau.edu.cn); [hainan@mail.hzau.edu.cn](mailto:hainan@mail.hzau.edu.cn);

3 The Key Laboratory of Aquatic Biodiversity and Conservation, Institute of Hydrobiology, Chinese Academy of Sciences, Wuhan 430072, China; [geyihao@ihb.ac.cn](mailto:geyihao@ihb.ac.cn); [wangjun168@ynu.edu.cn](mailto:wangjun168@ynu.edu.cn)

*Corresponding author: Qiong Zhou, College of Fisheries, Huazhong Agricultural University, Wuhan 430070, China.

Tel: +86-27 8728 2113. Fax: +86 27 8728 2114.

E-mail: [hainan@mail.hzau.edu.cn](mailto:hainan@mail.hzau.edu.cn)

**Table S1** Genetic distance analysis of Kimura-2-Parameter (K2P) for seven taxa of benthic macroinvertebrates.

| Taxa |  |  |  |  | K2P genetic distance (%) | | |
| --- | --- | --- | --- | --- | --- | --- | --- |
|  |  | N | Taxa | Comparisons | Minimum Maximum Mean and SD | | |
| Ephemeroptera | Within species | 500 | 37 | 5290 | 0.00 | 15.07 | 0.88±0.00 |
|  | Within genus | 360 | 11 | 5493 | 9.19 | 27.15 | 17.44±0.00 |
|  | Within family | 430 | 3 | 26086 | 16.84 | 31.34 | 22.17±0.00 |
| Plecoptera | Within species | 132 | 12 | 1432 | 0.00 | 3.41 | 0.89±0.00 |
|  | Within genus | 62 | 4 | 225 | 11.08 | 18.57 | 13.22±0.01 |
|  | Within family | 101 | 2 | 2985 | 16.10 | 23.88 | 18.85±0.01 |
| Trichoptera | Within species | 81 | 11 | 574 | 0.00 | 1.49 | 0.43±0.00 |
|  | Within genus | 41 | 2 | 252 | 11.81 | 22.37 | 14.39±0.01 |
|  | Within family | 61 | 2 | 801 | 16.39 | 24.75 | 19.28±0.00 |
| Diptera | Within species | 242 | 42 | 1108 | 0.00 | 7.26 | 0.48±0.00 |
|  | Within genus | 175 | 8 | 1780 | 0.62 | 33.36 | 14.40±0.00 |
|  | Within family | 190 | 4 | 12913 | 11.13 | 37.41 | 19.92±0.00 |
| Hemiptera | Within species | 61 | 8 | 405 | 0.00 | 2.33 | 0.57±0.00 |
|  | Within genus | 18 | 1 | 83 | 8.66 | 12.32 | 10.34±0.01 |
|  | Within family | 40 | 3 | 321 | 9.14 | 22.17 | 14.65±0.01 |
| Coleoptera | Within species | 47 | 11 | 134 | 0.00 | 2.46 | 0.63±0.00 |
|  | Within genus | 30 | 3 | 47 | 9.01 | 13.63 | 11.79±0.02 |
|  | Within family | 54 | 5 | 587 | 12.98 | 22.93 | 15.39±0.00 |
| Odonata | Within species | 21 | 4 | 49 | 0.00 | 1.10 | 0.28±0.01 |
|  | Within genus | 0 | 0 | 0 | 0 | 0 | 0 |
|  | Within family | 16 | 2 | 29 | 14.11 | 18.65 | 17.40±0.06 |
| All | Within species | 1085 | 125 | 9005 | 0.00 | 15.07 | 0.78±0.00 |
|  | Within genus | 689 | 29 | 8140 | 0.62 | 33.06 | 16.37±0.00 |
|  | Within family | 924 | 23 | 44146 | 9.14 | 37.41 | 21.08±0.00 |

**Table S2** High intraspecific divergence (ISD) led to the assignment of multiple BINs for conspecific individuals (83 individuals).

| **Taxa** | **Family** | **Species** | **BIN** | **N** | **Max ISD** |
| --- | --- | --- | --- | --- | --- |
| Ephemeroptera |  |  |  |  |  |
|  | Heptageniidae | Epeorus sp5 | BOLD:ADL2084 | 4 | 3.93 |
|  |  |  | BOLD:ADL2085 | 2 |  |
|  | Heptageniidae | Rhithrogena tianshanica | BOLD:ADL2320 | 13 | 3.15 |
|  |  |  | BOLD:ADL2319 | 1 |  |
|  | Ameletidae | Ameletus montanus | BOLD:ADL1468 | 23 | 15.07 |
|  |  |  | BOLD:ADL1469 | 4 |  |
| Diptera |  |  |  |  |  |
|  | Athericidae | Atherix sp. XJ | BOLD:ADL0095 | 5 | 3.44 |
|  |  |  | BOLD:ADL2450 | 1 |  |
|  | Chironomidae | Glyptotendipes sp. XJ | BOLD:ADL0144 | 5 | 5.04 |
|  |  |  | BOLD:ACD4471 | 1 |  |
|  | Chironomidae | Euryhapsis sp | BOLD:ADL0322 | 3 | 4.44 |
|  |  |  | BOLD:ADL0672 | 8 |  |
|  |  |  | BOLD:ADL1075 | 1 |  |
|  |  |  | BOLD:ADL1506 | 1 |  |
|  | Chironomidae | Dicranota guerini | BOLD:ADL1566 | 1 | 7.26 |
|  |  |  | BOLD:ADL1665 | 1 |  |
|  |  |  | BOLD:ADL2282 | 5 |  |
|  | Chironomidae | Cricotopus ornatus | BOLD:AAP5926 | 2 | 2.34 |
|  |  |  | BOLD:ADL1458 | 2 |  |


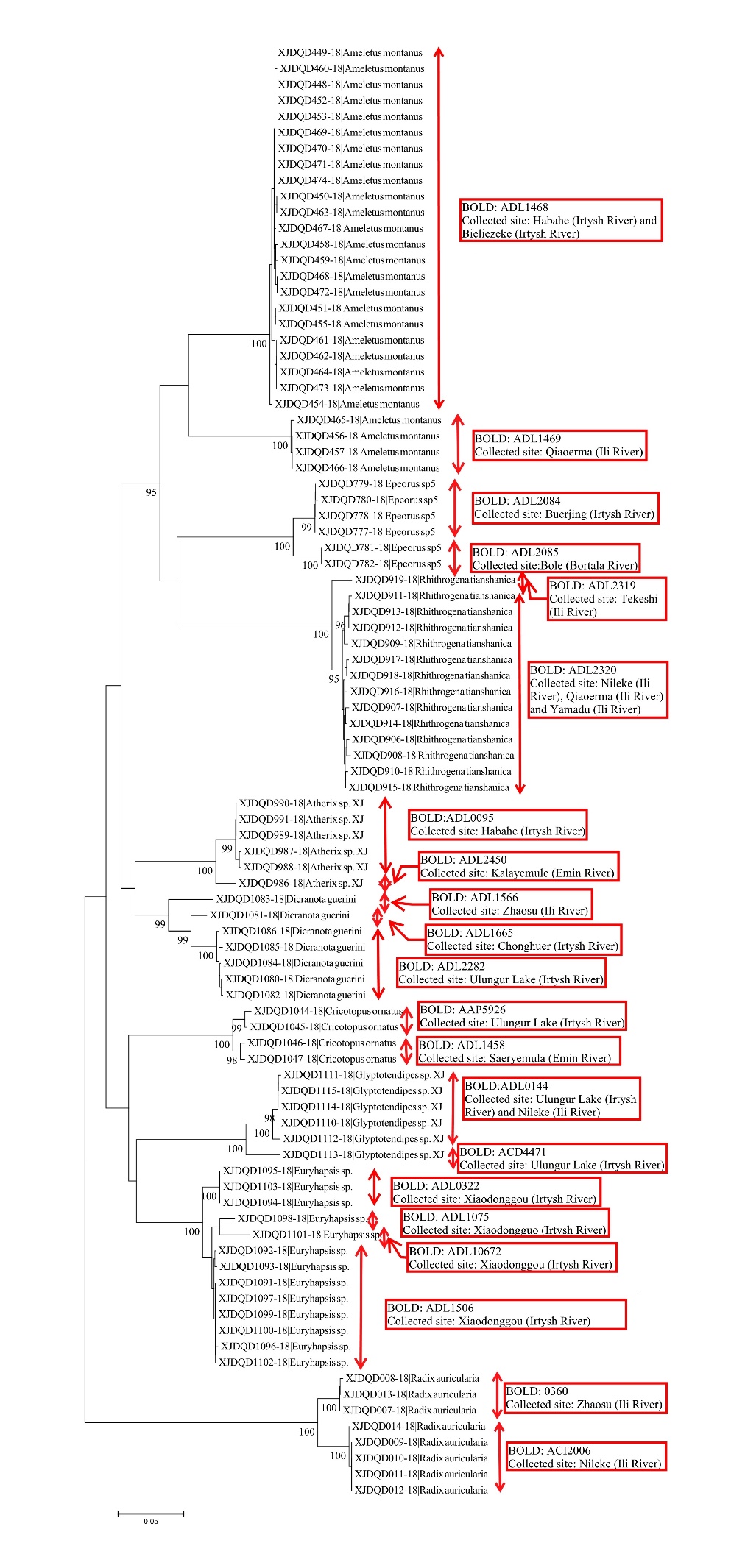


**Fig. S1** The nine species with large internal splits in phylogenetic tree.


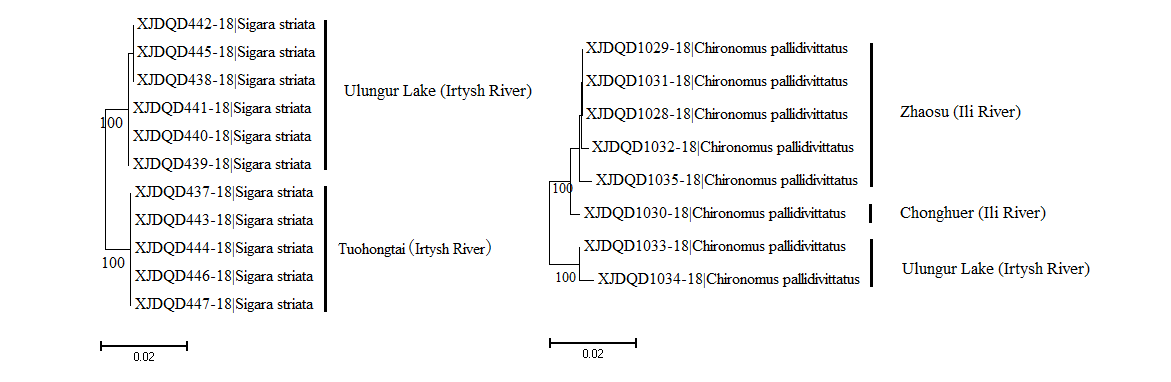


**Fig. S2** The NJ tree of *Sigara striata* and *Chironomus pallidivittatus*.

**Part I**

**Species sequences from other areas in the BOLD database.**

| Order | ID | Sampling sites | References |
| --- | --- | --- | --- |
| Hemiptera | FBAQU016-09\|Sigara_falleni\|COI-5P\|GU682186 | Germany, Bavaria, Salzach-Huegelland | Raupach et al., 2014 |
|  | FBAQU028-09\|Notonecta_glauca\|COI-5P\|HM376124 | Germany, Bavaria, Salzach-Huegelland |  |
|  | FBAQU1298-12\|Sigara_semistriata\|COI-5P\|KM022152 | Germany, Bavaria, Salzach-Huegelland |  |
|  | FBAQU162-09\|Sigara_falleni\|COI-5P\|GU682177 | Germany, Bavaria, Steigerwald |  |
|  | FBAQU164-09\|Sigara_lateralis\|COI-5P\|HQ948123 | Germany, Bavaria, Steigerwald |  |
|  | FBAQU210-09\|Sigara_fossarum\|COI-5P\|HM421994 | Germany, Bavaria, Hinterer Bayerischer Wald |  |
|  | FBAQU211-09\|Sigara_striata\|COI-5P\|HM421995 | Germany, Bavaria, Mittelfraenkisches Becken |  |
|  | FBAQU708-10\|Notonecta_glauca\|COI-5P\|HQ563136 | Germany, Bavaria, Donau-Isar-Huegelland |  |
|  | FBAQU203-09\|Callicorixa praeusta\|COI-5P\|HM421991 | Germany, Bavaria, Ammer-Loisach-Huegelland |  |
|  | FBAQU709-10\|Notonecta_lutea\|COI-5P\|HQ563137 | Germany, Bavaria, Inn-Chiemsee-Huegelland |  |
|  | FBAQU710-10\|Notonecta_maculata\|COI-5P\|HQ563138 | Germany, Bavaria, Donau-Isar-Huegelland |  |
|  | FBHET1045-12\|Notonecta_glauca\|COI-5P\|KM023116 | Germany, Mecklenburg-Vorpommern, Luhme |  |
|  | FBAQU1274-12\|Hesperocorixa linnaei\|COI-5P\|KM02231 | Germany, Bavaria, Inn-Chiemsee-Huegelland |  |
|  | FBAQU014-09\|Ilyocoris cimicoides\|COI-5P\|HM376118 | Germany, Bavaria, Salzach-Huegelland |  |
|  | FBAQU209-09\|Nepa cinerea\|COI-5P\|HM421993 | Germany, Bavaria, Ammer-Loisach-Huegelland |  |
|  | FBAQU1291-12\|Paracorixa concinna\|COI-5P\|KM023136 | Germany, Bavaria, Ammer-Loisach-Huegelland |  |
|  | FBAQU711-10\|Ranatra linearis\|COI-5P\|HQ563139 | Germany, Bavaria, Falkensteiner Vorwald |  |
|  | FBAQU181-09\|Ranatra linearis\|COI-5P\|HM376198 | Germany, Bavaria, Mittelfraenkisches Becken |  |
|  | FBAQU1292-12\|Sigara falleni\|COI-5P\|KM021936 | Germany, Bavaria, Donauried |  |
| Ephemeroptera | EVOTR030-12\|Ameletus_celer\|COI-5P | United States, Colorado | — |
|  | GBA19851-14\|Baetis_canariensis\|COI-5P\|KF438148 | Mined from GenBank, NCBI | — |
|  | LJMAY309-11\|Baetis_magnus\|COI-5P\|JQ662487 | Mexico, Veracruz, unnamed stream | — |
|  | BKMAY026-11\|Baetis_magnus\|COI-5P\|JQ662082 | United States, Colorado, Larimer County | — |
|  | ABSTO030-09\|Baetis_bicaudatus\|COI-5P\|GU711490 | Canada, Alberta, Banff NP | — |
|  | ANCN130-10\|Baetis_bicaudatus\|COI-5P\|KM530609 | Canada, Alberta, Waterton Lakes NP | — |
|  | BBEPT050-10\|Baetis_bicaudatus\|COI-5P\|JN291743 | Canada, Alberta, Jasper NP | — |
|  | BBEPT098-10\|Baetis_bicaudatus\|COI-5P\|JN291758 | Canada, British Columbia, Kootenay NP | — |
|  | EVOTR213-12\|Baetis_bicaudatus\|COI-5P | United States, Colorado | — |
|  | GBMH0768-06\|Baetis_bicaudatus\|COI-5P\|AY383574 | Mined from GenBank, NCBI | — |
|  | SSJAD1568-13\|Baetis_bicaudatus\|COI-5P\|KM537228 | Canada, Alberta, Jasper NP | — |
|  | CFWIA725-10\|Baetis_sp._CA1\|COI-5P\|HQ938900 | United States, California, Los Angeles | Stein et al., 2013 |
|  | EPHFI107-12\|Caenis_horaria\|COI-5P\|KC158559 | Norway, Finnmark, Sor-Varanger | Kjaerstad et al., 2012 |
|  | ABMAY009-09\|Cinygmula_sp.JMW4\|COI-5P\|JQ661644 | Canada, Alberta, Waterton Lakes NP | — |
|  | ABMAY039-09\|Cinygmula_spJMW3\|COI-5P\|HM372922 | Canada, Alberta, Waterton Lakes NP | — |
|  | BKMAY047-11\|Cinygmula_mimus\|COI-5P\|JQ663303 | United States, Colorado, Larimer County | — |
|  | EVOTR306-12\|Cinygmula_sp._B_BG\|COI-5P | United States, Colorado | — |
|  | DHF363-10\|Drunella_lata\|COI-5P\|HM399272 | United States, Pennsylvania | — |
|  | GBA17274-14\|Epeorus_latifolium\|COI-5P\|KF563019 | Japan | — |
|  | GBMIN31030-13\|Epeorus_latifolium\|COI-5P\|AB538378 | Japan, Nagano | — |
|  | LJGSM062-10\|Ephemera blanda\|COI-5P\|HQ571211 | United States, North Carolina, Swain Co. | — |
|  | HIMXD114-10\|Ephemera varia\|COI-5P\|HQ958737 | United States, North Carolina, Swain Co. | — |
|  | GBA17277-14\|Ephemerella_atagosana\|COI-5P\|KF563038 | Japan | — |
|  | GBMIN26021-13\|Serratella_ignita\|COI-5P\|JN164273 | Mined from GenBank, NCBI | — |
|  | EPHFI036-11\|Ephemerella_aurivillii\|COI-5P\|JN299102 | Norway, Finnmark, Porsanger | Kjaerstad et al., 2012 |
|  | AMIG046-08\|Siphlonurus_quebecensis\|COI-5P\|HQ151910 | United States, Maryland | Erik et al., 2011 |
|  | ABMAY012-09\|Baetis persecutor\|COI-5P\|JQ661728 | Canada, Alberta, Waterton Lakes NP | — |
|  | ABSTO033-09\|Baetis bicaudatus\|COI-5P\|GU711491 | Canada, Alberta, Banff NP | — |
|  | BKMAY061-11\|Baetis phoebus\|COI-5P\|JQ663353 | United States, Colorado, Boulder County | — |
|  | BKMAY062-11\|Baetis phoebus\|COI-5P\|JQ662027 | United States, Colorado, Boulder County | — |
|  | BKMAY063-11\|Baetis phoebus\|COI-5P\|JQ663043 | United States, Colorado, Boulder County | — |
|  | FBAQU1332-13\|Caenis lactea\|COI-5P\|KY261531 | Germany, Bavaria, South Bavaria | — |
|  | EPHFI037-11\|Ephemerella aurivillii\|COI-5P\|JN299103 | Norway, Finnmark, Porsanger | Kjaerstad et al., 2012 |
|  | EPHFI038-11\|Ephemerella aurivillii\|COI-5P\|JN299104 | Norway, Finnmark, Alta | Kjaerstad et al., 2012 |
|  | BGMAY003-10\|Ephemerella mucronata\|COI-5P | Bulgaria, Sofiya, Kyustendil | — |
|  | EPHFI063-11\|Ephemerella mucronata\|COI-5P\|JN299127 | Norway, Finnmark, Kautokeino | Kjaerstad et al., 2012 |
|  | EPHFI064-11\|Ephemerella mucronata\|COI-5P\|JN299128 | Norway, Finnmark, Kautokeino | Kjaerstad et al., 2012 |
|  | EPHFI048-11\|Heptagenia dalecarlica\|COI-5P\|JN299113 | Norway, Finnmark, Alta | Kjaerstad et al., 2012 |
|  | EPHFI049-11\|Heptagenia dalecarlica\|COI-5P\|JN299114 | Norway, Finnmark, Alta | Kjaerstad et al., 2012 |
|  | EPHFI050-11\|Heptagenia dalecarlica\|COI-5P\|JN299115 | Norway, Finnmark, Alta | Kjaerstad et al., 2012 |
|  | FBAQU1107-12\|Heptagenia flava\|COI-5P\|KY262334 | Germany, Oberpfalz | — |
|  | FBAQU830-10\|Heptagenia flava\|COI-5P\|HQ563234 | Germany, Bavaria, Suedliche Frankenalb | — |
|  | FBAQU831-10\|Heptagenia flava\|COI-5P\|HQ563235 | Germany, Bavaria, Suedliche Frankenalb | — |
| Plecoptera | CRBC001-13\|Pteronarcys_californica\|COI-5P | Canada, British Columbia | — |
|  | GBMH1029-06\|Pteronarcys_princeps\|COI-5P\|AY687866 | Mined from GenBank, NCBI | — |
|  | GBMH8736-13\|Capnia_vidua\|COI-5P\|JQ736349 | Italy | — |
|  | AMIA013-07\|Pteronarcys\|COI-5P\|HQ150591 | United States, Maryland, AL | Erik et al., 2011 |
|  | MBSS035-08\|Taenionema_atlanticum\|COI-5P\|HQ152782 | United States, Maryland | Erik et al., 2011 |
|  | MDA081-08\|Agnetina_flavescens\|COI-5P\|JN200441 | United States, Virginia, Fairfax Co | — |
|  | MDA082-08\|Agnetina_annulipes\|COI-5P\|JN200437 | United States, Virginia, Fairfax Co | — |
|  | MDA693-09\|Paracapnia_angulata\|COI-5P\|JN200674 | United States, Pennsylvania, Forest Co | — |
|  | MDA694-09\|Paracapnia_angulata\|COI-5P\|JN200675 | United States, Pennsylvania, Forest Co | — |
|  | MDA797-09\|Agnetina annulipes\|COI-5P\|JN200438 | United States, Virginia, Fairfax Co | — |
|  | MDA801-09\|Agnetina capitata\|COI-5P\|JN200439 | United States, Virginia, Smyth Co | — |
|  | PLENB001-09\|Agnetina capitata\|COI-5P\|KR148211 | Canada, New Brunswick, Fredericton | — |
|  | PLENB005-09\|Agnetina capitata\|COI-5P\|KR144479 | Canada, New Brunswick, Fredericton | — |
|  | NOEPT041-11\|Amphinemura borealis\|COI-5P\|JX495646 | Norway, Troms, Skibotn | — |
|  | NOEPT053-11\|Amphinemura borealis\|COI-5P\|JX495640 | Norway, Finnmark, Alta | — |
|  | GBMIX1862-15\|Amphinemura borealis\|COI-5P\|KY262067 | Germany, Bavaria, Buchenhain | — |
|  | GBMIN12330-13\|Arcynopteryx compacta\|COI-5P\|JF312860 | Mined from GenBank, NCBI | — |
|  | GBMIN12370-13\|Arcynopteryx compacta\|COI-5P\|JF312861 | Mined from GenBank, NCBI | — |
|  | UAMIC779-13\|Arcynopteryx compacta\|COI-5P\|KU874207 | United States, Alaska | — |
|  | GBMIN18722-13\|Besdolus ravizzarum\|COI-5P\|JN034539 | Mined from GenBank, NCBI | — |
|  | SMTPL6337-15\|Capnia vernalis\|COI-5P\|MG381075 | Canada, Alberta, Drayton Valley | — |
|  | SMTPL6336-15\|Capnia vernalis\|COI-5P\|MG383077 | Canada, Alberta, Drayton Valley | — |
|  | GBEPT1805-14\|Diura bicaudata\|COI-5P\|KY262389 | Germany, Bavaria | — |
|  | AMIG194-08\|Isoperla dicala\|COI-5P\|HQ151954 | United States, Maryland, QA | Erik et al., 2011 |
|  | AMIE176-07\|Isoperla sp. CNW\|COI-5P\|HQ151457 | United States, Maryland, SM | Erik et al., 2011 |
|  | AMIA120-07\|Isoperla\|COI-5P\|HQ150501 | United States, Maryland, QA | Erik et al., 2011 |
|  | FBAQU037-09\|Nemoura cinerea\|COI-5P\|GU682181 | Germany, Bavaria, Falkensteiner Vorwald | — |
|  | GBMIN18729-13\|Perlodes microcephalus\|COI-5P\|JN034552 | Mined from GenBank, NCBI | — |
|  | GBMIN18718-13\|Perlodes microcephalus\|COI-5P\|JN034549 | Mined from GenBank, NCBI | — |
|  | AMIJ013-08\|Pteronarcys\|COI-5P\|HQ152612 | United States, Maryland | Erik et al., 2011 |
|  | AMIA147-07\|Pteronarcys\|COI-5P\|HQ150590 | United States, Maryland, AL | Erik et al., 2011 |
|  | AMIJ118-08\|Pteronarcys proteus\|COI-5P\|HQ152611 | United States, Maryland | Erik et al., 2011 |
|  | GBMH3808-08\|Siphonoperla montana\|COI-5P\|EU441197 | Mined from GenBank, NCBI | — |
|  | GBMIN29109-13\|Siphonoperla montana\|COI-5P\|HQ705632 | Mined from GenBank, NCBI | — |
|  | EVOTR1284-12\|Taenionema pallidum\|COI-5P | United States, Colorado | — |
|  | CNGCA033-15\|Taenionema pallidum\|COI-5P\|MG468363 | Canada, British Columbia, Glacier National Park | — |
|  | BKSTO429-11\|Taenionema pallidum\|COI-5P | United States, Wyoming, Johnson County | — |
| Trichoptera | BBEPT434-11\|Ceratopsyche_alternans\|COI-5P\|KM535763 | Canada, Saskatchewan, Prince Albert NP | — |
|  | BBEPT564-11\|Brachycentrus_americanus\|COI-5P\|KM532088 | Canada, Alberta, Jasper NP | — |
|  | BBEPT667-11\|Potamyia_flava\|COI-5P\|KM536428 | Canada, Ontario, Point Pelee NP | — |
|  | BBHYE495-10\|Ceratopsyche_alternans\|COI-5P\|HQ929276 | Canada, Nova Scotia, Kejimkujik NP | — |
|  | EVCAD564-07\|Brachycentrus_americanus\|COI-5P\|HM137758 | Canada, New Brunswick | — |
|  | GBMH6364-09\|Brachycentrus_americanus\|COI-5P\|AF436503 | Mined from GenBank, NCBI | — |
|  | GBMH7754-10\|Potamyia_elektra\|COI-5P\|FM998449 | Mined from GenBank, NCBI | — |
|  | GBMIN18120-13\|Ceratopsyche_kozhantschikovi\|COI-5P\|KC135956 | South Korea | — |
|  | MDA855-09\|Brachycentrus_americanus\|COI-5P\|JN200476 | United States, Iowa, Winneshiek Co. | — |
|  | MGCAD036-08\|Arctopsyche amurensis\|COI-5P\|KX10568 | United States, Wyoming, Johnson County | Zhou et al., 2016 |
|  | MGCAD037-08\|Arctopsyche amurensis\|COI-5P\|KX106589 | Mongolia, Tov, Erdene | Zhou et al., 2016 |
|  | MGCAD038-08\|Arctopsyche amurensis\|COI-5P\|KX104478 | Mongolia, Tov, Erdene | Zhou et al., 2016 |
|  | ABCAD049-08\|Brachycentrus americanus\|COI-5P\|KM529085 | Canada, Alberta, Waterton Lakes NP | Zhou et al., 2016 |
|  | ABCAD073-08\|Brachycentrus americanus\|COI-5P\|KM533621 | Canada, Alberta, Banff NP | Zhou et al., 2016 |
|  | ABCAD079-08\|Brachycentrus americanus\|COI-5P\|KM535119 | Canada, Alberta, Banff NP | Zhou et al., 2016 |
|  | RMCAD504-08\|Dicosmoecus obscuripennis\|COI-5P\|HM382243 | Canada, Yukon Territory | Zhou et al., 2016 |
|  | FBAQU058-09\|Ecnomus tenellus\|COI-5P\|GU713208 | Germany, Bavaria, Donaumoos | Zhou et al., 2016 |
|  | FBAQU111-09\|Ecnomus tenellus\|COI-5P\|GU713194 | Germany, Bavaria, Ammer-Loisach-Huegelland | Zhou et al., 2016 |
|  | GBEPT782-14\|Ecnomus tenellus\|COI-5P\|KX292881 | Germany, Brandenburg | Zhou et al., 2016 |
|  | HMKKT023-10\|Glossosoma minutum\|COI-5P\|HQ958934 | Kyrgyzstan, Naryn | Zhou et al., 2016 |
|  | ESCAD019-10\|Halesus tessellatus\|COI-5P\|HQ561930 | Spain, Andalusia, Jaen | Zhou et al., 2016 |
|  | ESCAD020-10\|Halesus tessellatus\|COI-5P\|HQ561931 | Spain, Andalusia, Jaen | Zhou et al., 2016 |
|  | FBAQU1177-12\|Halesus tessellatus\|COI-5P\|KX294388 | Germany, Bavaria, Isar-Inn-Huegelland | Zhou et al., 2016 |
|  | HMKKT987-11\|Hydropsyche demavenda\|COI-5P\|KX141528 | Kyrgyzstan, Batken, Batken | Zhou et al., 2016 |
|  | CAUTR017-09\|Hydropsyche kozhantschikovi\|COI-5P\|HM405370 | China, Beijing Shi, Mi-yun | Zhou et al., 2016 |
|  | CAUTR018-09\|Hydropsyche kozhantschikovi\|COI-5P\|HM405371 | China, Beijing Shi, Mi-yun | Zhou et al., 2016 |
|  | CAUTR020-09\|Hydropsyche kozhantschikovi\|COI-5P\|HM405373 | China, Beijing Shi, Mi-yun | Zhou et al., 2016 |
|  | JPCAD441-08\|Hydropsyche ornatula\|COI-5P\|KX103522 | Hungary, Somogy | Zhou et al., 2016 |
|  | MGCAD119-08\|Hydropsyche ornatula\|COI-5P\|KX104965 | Mongolia, Selenge, Zuunburen sum | Zhou et al., 2016 |
|  | BHMKK204-12\|Hydropsyche pellucidula\|COI-5P\|KX295403 | Italy, S. Albano Stura CN, Stura di Demonte river | Zhou et al., 2016 |
|  | BHMKK216-12\|Hydropsyche pellucidula\|COI-5P\|KX294342 | Italy, Casale Monferrato AL, Terranove | Zhou et al., 2016 |
|  | BHMKK227-12\|Hydropsyche pellucidula\|COI-5P\|KX293565 | Austria, Upper Austria, St. Florian | Zhou et al., 2016 |
|  | MGCAD130-08\|Hydropsyche valvata\|COI-5P\|KX102929 | Mongolia, Hovsgol | Zhou et al., 2016 |
|  | MGCAD147-08\|Hydropsyche valvata\|COI-5P\|KX103394 | Mongolia, Bulgan, Teshig | Zhou et al., 2016 |
|  | HMCAD479-09\|Potamyia baenzigeri\|COI-5P\|KX107510 | Thailand, Lampang, Chaeson National Park | Zhou et al., 2016 |
|  | HMCAD480-09\|Potamyia baenzigeri\|COI-5P\|KX102899 | Thailand, Lampang, Chaeson National Park | Zhou et al., 2016 |
|  | CRTRI033-15\|Rhyacophila arnaudi\|COI-5P\|KX144427 | Canada, British Columbia | Zhou et al., 2016 |
|  | CRTRI085-15\|Rhyacophila arnaudi\|COI-5P\|KX140705 | Canada, British Columbia | Zhou et al., 2016 |
|  | CRTRI102-15\|Rhyacophila arnaudi\|COI-5P\|KX140935 | Canada, British Columbia | Zhou et al., 2016 |
|  | RUSST110-12\|Stenopsyche marmorata\|COI-5P\|KX293423 | Russia, Primorskiy Kray, Khasanskyi | Zhou et al., 2016 |
|  | RUSST313-12\|Stenopsyche marmorata\|COI-5P\|KX294466 | Russia, Primorskiy Kray, Ussuri River | Zhou et al., 2016 |
| Coleoptera | GCOL10897-16\|Agabus biguttatus\|COI-5P\|KU918758 | Germany, Thuringia, Solkwitz | — |
|  | GCOL12451-16\|Agabus biguttatus\|COI-5P\|KU913456 | Germany, Saxony-Anhalt, Halberstadt | — |
|  | GCOL12452-16\|Agabus biguttatus\|COI-5P\|KU907877 | Germany, Saxony-Anhalt, Halberstadt | — |
|  | UAMIC042-12\|Bembidion gratiosum\|COI-5P\|KU874331 | United States, Alaska | — |
|  | UAMIC370-13\|Bembidion gratiosum\|COI-5P\|KU874330 | United States, Alaska | — |
|  | FBCOE1227-12\|Berosus frontifoveatus\|COI-5P\|KM443487 | Germany, Rhineland-Palatinate, Noerdliche | — |
|  | GCOL054-16\|Berosus frontifoveatus\|COI-5P\|KU908671 | Germany, Rhineland-Palatinate, Buechelberg | — |
|  | GCOL3596-16\|Berosus frontifoveatus\|COI-5P\|KU908154 | Germany, Rhineland-Palatinate, Buechelberg | — |
|  | GCOL4163-16\|Colymbetes fuscus\|COI-5P\|KU914464 | Germany, Thuringia, Werratal | — |
|  | GCOL837-16\|Colymbetes fuscus\|COI-5P\|KU917492 | France, Brecy-Brieres | — |
|  | FBAQU154-09\|Elmis rioloides\|COI-5P\|HM376183 | Germany, Bavaria, Regensenke | — |
|  | FBAQU405-10\|Elmis rioloides\|COI-5P\|HM401304 | Germany, Bavaria, Inn-Chiemsee-Huegelland | — |
|  | FBCOD262-11\|Graphoderus austriacus\|COI-5P\|KM447476 | Germany, Mecklenburg-Vorpommern | — |
|  | FBCOD263-11\|Graphoderus austriacus\|COI-5P\|KM446245 | Germany, Mecklenburg-Vorpommern | — |
|  | GCOL890-16\|Graphoderus austriacus\|COI-5P\|KU909812 | Austria, Illmitz | — |
|  | COLFD546-12\|Gyrinus marinus\|COI-5P\|KJ964209 | Finland, Savonia australis | Pentinsaari et al., 2014 |
|  | GCOL8888-16\|Gyrinus marinus\|COI-5P\|KU913511 | Germany, North Rhine-Westphalia | Pentinsaari et al., 2014 |
|  | GCOL988-16\|Gyrinus marinus\|COI-5P\|KU913593 | Germany, North Rhine-Westphalia | Pentinsaari et al., 2014 |
|  | COLFA387-12\|Gyrinus opacus\|COI-5P\|KJ963052 | Finland, Northern Ostrobothnia | Pentinsaari et al., 2014 |
|  | COLFD017-12\|Gyrinus opacus\|COI-5P\|KJ964534 | Finland, Lapland | Pentinsaari et al., 2014 |
|  | COLFB180-12\|Ilybius crassus\|COI-5P\|KJ965207 | Finland, Northern Ostrobothnia | Pentinsaari et al., 2014 |
|  | COLFB181-12\|Ilybius crassus\|COI-5P\|KJ965439 | Finland, Northern Ostrobothnia | Pentinsaari et al., 2014 |
|  | COLFF072-13\|Ilybius crassus\|COI-5P\|KJ966540 | Finland, Regio kuusamoensis | Pentinsaari et al., 2014 |
|  | COLFD247-12\|Laccophilus minutus\|COI-5P\|KJ963826 | Estonia | Pentinsaari et al., 2014 |
|  | COLFE206-12\|Laccophilus minutus\|COI-5P\|KJ965836 | Finland, Regio aboensis | Pentinsaari et al., 2014 |
|  | GCOL3763-16\|Laccophilus minutus\|COI-5P\|KU906692 | Germany, North Rhine-Westphalia, Bornheim | Pentinsaari et al., 2014 |
|  | FBAQU441-10\|Laccophilus poecilus\|COI-5P\|HM401334 | Germany, Bavaria, Inn-Chiemsee-Huegelland | Pentinsaari et al., 2014 |
|  | FBAQU969-10\|Laccophilus poecilus\|COI-5P\|JF889434 | Germany, Mecklenburg-Vorpommern | Pentinsaari et al., 2014 |
|  | COAS548-12\|Mecyclothorax\|COI-5P | Australia, Australian Capital Territory, Canberra | Pentinsaari et al., 2014 |
|  | COAS716-12\|Mecyclothorax\|COI-5P | Australia, Australian Capital Territory | Pentinsaari et al., 2014 |
|  | GBCL24705-15\|Nebrioporus baeticus\|COI-5P\|HF947990 | Mined from GenBank, NCBI | — |
|  | GBCL24706-15\|Nebrioporus baeticus\|COI-5P\|HF947991 | Mined from GenBank, NCBI | — |
|  | COLFB013-12\|Noterus clavicornis\|COI-5P\|KJ965723 | Finland, Aland Islands, Alandia | Pentinsaari et al., 2014 |
|  | COLFB015-12\|Noterus clavicornis\|COI-5P\|KJ963775 | Finland, Aland Islands, Alandia | Pentinsaari et al., 2014 |
|  | FBAQU095-09\|Noterus clavicornis\|COI-5P\|HM401273 | Germany, Bavaria, Donaumoos | Pentinsaari et al., 2014 |
|  | GCOL805-16\|Orectochilus villosus\|COI-5P\|KU909801 | Germany, North Rhine-Westphalia, Nordrhein | — |
|  | FBAQU976-10\|Rhantus notatus\|COI-5P\|JF889436 | Germany, Mecklenburg-Vorpommern | — |
|  | FBAQU997-10\|Rhantus notatus\|COI-5P\|JF889447 | Germany, Mecklenburg-Vorpommern | — |
|  | FBAQU998-10\|Rhantus notatus\|COI-5P\|JF889448 | Germany, Mecklenburg-Vorpommern | — |
|  | COAS1028-12\|Rhantus suturalis\|COI-5P | Australia, Australian Capital Territory, Canberra | — |
|  | COAS846-12\|Rhantus suturalis\|COI-5P | Australia, Australian Capital Territory, Canberra | — |
|  | COLFE222-12\|Rhantus suturalis\|COI-5P\|KJ963022 | Finland, Regio aboensis | Pentinsaari et al., 2014 |
|  | BBCCA2973-12\|Stenelmis crenata\|COI-5P | United States, Florida, Santa Rosa County | Pentinsaari et al., 2014 |
|  | BBCEC498-10\|Stenelmis crenata\|COI-5P\|HQ551637 | Canada, Newfoundland and Labrador | Pentinsaari et al., 2014 |
| Odonata | GBMIN88590-17\|Coenagrion puella\|COI-5P | Portugal | — |
|  | ODOPH109-13\|Coenagrion pulchellum\|COI-5P\|KF369349 | Ukraine | — |
|  | FBAQU015-09\|Enallagma cyathigerum\|COI-5P\|GU682183 | Germany, Bavaria, Salzach-Huegelland | — |
|  | FBAQU1504-13\|Enallagma cyathigerum\|COI-5P | Germany, Brandenburg | — |
|  | ODRMA077-10\|Enallagma cyathigerum\|COI-5P\|JF839291 | Canada, Yukon Territory, Central Yukon | — |
|  | GBMIN88687-17\|Gomphus flavipes\|COI-5P | Mined from GenBank, NCBI | — |
|  | GBMH7974-10\|Ischnura elegans\|COI-5P\|GQ256031 | Mined from GenBank, NCBI | — |
|  | GBMIN88606-17\|Ischnura elegans\|COI-5P | Mined from GenBank, NCBI | — |
|  | GBMIN88687-17\|Gomphus flavipes\|COI-5P | Mined from GenBank, NCBI | — |
|  | FBAQU1444-13\|Sympecma paedisca\|COI-5P | Germany, Bavaria | — |
|  | GBMHO553-14\|Sympecma paedisca\|COI-5P\|KF257126 | Mined from GenBank, NCBI | — |
|  | GBMIN88603-17\|Ischnura elegans\|COI-5P | Mined from GenBank, NCBI | — |
| Diptera | GBDP9433-12\|Chaoborus flavicans\|COI-3P\|AJ427617 | Mined from GenBank, NCBI | — |
|  | GKBRA194-16\|Chaoborus flavicans\|COI-5P | Germany, Schleswig-Holstein, Westensee | — |
|  | BSCHI100-11\|Chironomus annularius\|COI-5P\|KC250747 | Sweden, Uppland | Y. Brodin et al., 2012 |
|  | GBDP0492-06\|Chironomus annularius\|COI-5P\|AF192189 | Mined from GenBank, NCBI | Y. Brodin et al., 2012 |
|  | BSCHI065-11\|Chironomus pallidivittatus\|COI-5P\|KC250749 | Sweden, Uppland | Y. Brodin et al., 2012 |
|  | BSCHI066-11\|Chironomus pallidivittatus\|COI-5P\|KC250750 | Sweden, Uppland | Y. Brodin et al., 2012 |
|  | BSCHI009-11\|Cricotopus bicinctus\|COI-LIKE | Finland, Aland Islands, Aland | Y. Brodin et al., 2012 |
|  | BSCHI009-11\|Cricotopus bicinctus\|COI-5P\|KC250773 | Finland, Aland Islands, Aland | Y. Brodin et al., 2012 |
|  | BSCHI010-11\|Cricotopus bicinctus\|COI-5P\|KC250771 | Finland, Aland Islands, Aland | Y. Brodin et al., 2012 |
|  | BSCHI022-11\|Cricotopus bicinctus\|COI-5P\|KC250772 | Sweden, Skane | Y. Brodin et al., 2012 |
|  | ARCHR078-11\|Cricotopus ornatus\|COI-5P\|KC130766 | Norway, Finnmark, Sor-Varanger | Susan et al., 2012 |
|  | ARCHR082-11\|Cricotopus ornatus\|COI-5P\|KC130791 | Norway, Finnmark, Sor-Varanger | Susan et al., 2012 |
|  | BSCHI018-11\|Cricotopus ornatus\|COI-5P\|KC250779 | Sweden, Skane | Susan et al., 2012 |
|  | BBDIQ194-10\|Cricotopus sylvestris\|COI-5P\|HQ552212 | Canada, Ontario, Kawartha | — |
|  | BSCHI001-11\|Cricotopus sylvestris\|COI-5P\|KC250788 | Finland, Aland Islands, Aland | — |
|  | BSCHI002-11\|Cricotopus sylvestris\|COI-5P\|KC250789 | Finland, Aland Islands, Aland | — |
|  | BSCHI174-17\|Cricotopus triannulatus\|COI-5P | Sweden, Aangermanland | — |
|  | BSCHI321-17\|Cricotopus triannulatus\|COI-5P | Sweden, Gotland | — |
|  | BSCHI464-17\|Cricotopus triannulatus\|COI-5P | Sweden, Gotland | — |
|  | ASDMT233-11\|Cricotopus trifascia\|COI-5P\|MG142621 | Canada, Ontario, Guelph | — |
|  | BARSL054-16\|Cricotopus trifascia\|COI-5P | Canada, Ontario, Kawartha Lakes | — |
|  | BBDIT1703-12\|Cricotopus trifascia\|COI-5P | United States, Arizona, Pinal County | — |
|  | ASDMT233-11\|Cricotopus trifascia\|COI-5P\|MG142621 | Canada, Ontario, Guelph | — |
|  | BARSL054-16\|Cricotopus trifascia\|COI-5P | Canada, Ontario, Kawartha Lakes | — |
|  | GBMIN55289-17\|Diamesa tonsa\|COI-5P | Mined from GenBank, NCBI | — |
|  | GBMIN55290-17\|Diamesa tonsa\|COI-5P | Mined from GenBank, NCBI | — |
|  | GBMIN55291-17\|Diamesa tonsa\|COI-5P | Mined from GenBank, NCBI | — |
|  | BSCHR111-10\|Dicranota guerini\|COI-5P\|HQ937747 | Canada, Manitoba, Churchill | — |
|  | FINTI073-11\|Dicranota guerini\|COI-5P | Finland, Lapland, Lapponia enontekiensis | — |
|  | FINTI106-11\|Dicranota guerini\|COI-5P | Finland, Lapland, Lapponia enontekiensis | — |
|  | BSCHI380-17\|Endochironomus tendens\|COI-5P | Sweden, Aangermanland | — |
|  | BSCHI636-17\|Endochironomus tendens\|COI-5P | Sweden, Smaaland | — |
|  | BSCHI666-17\|Endochironomus tendens\|COI-5P | Poland, Zachodniopomorskie | — |
|  | GBDPC315-14\|Lipiniella moderata\|COI-5P\|AB838668 | Japan | — |
|  | GBMIN34272-13\|Orthocladius nitidoscutellatus\|COI-5P\|JF764777 | Iran, Asara | — |
|  | GMGMF382-14\|Orthocladius nitidoscutellatus\|COI-5P | Germany, Rhineland-Palatinate, Kreis Ahrweiler | — |
|  | GMGMF936-14\|Orthocladius nitidoscutellatus\|COI-5P | Germany, Rhineland-Palatinate, Kreis Ahrweiler | — |
|  | GMGMF094-14\|Orthocladius thienemanni\|COI-5P | Germany, Rhineland-Palatinate, Kreis Ahrweiler | — |
|  | GMGMF1334-14\|Orthocladius thienemanni\|COI-5P | Germany, Rhineland-Palatinate, Kreis Ahrweiler | — |
|  | GMGMF1363-14\|Orthocladius thienemanni\|COI-5P | Germany, Rhineland-Palatinate, Kreis Ahrweiler | — |
|  | BSCHI090-11\|Polypedilum nubeculosum\|COI-5P\|KC250832 | Sweden, Uppland | Y. Brodin et al., 2012 |
|  | BSCHI580-17\|Polypedilum nubeculosum\|COI-5P | Sweden, Haelsingland | Y. Brodin et al., 2012 |
|  | GBMIN55211-17\|Polypedilum nubeculosum\|COI-5P | China | Y. Brodin et al., 2012 |
|  | BSCHI083-11\|Procladius crassinervis\|COI-5P\|KC250836 | Sweden, Uppland | Y. Brodin et al., 2012 |
|  | BSCHI084-11\|Procladius crassinervis\|COI-5P\|KC250835 | Sweden, Uppland | Y. Brodin et al., 2012 |
|  | BSCHI222-17\|Procladius crassinervis\|COI-5P | Sweden, Uppland | Y. Brodin et al., 2012 |
|  | BFUKP049-12\|Simulium equinum\|COI-5P | United Kingdom, Norfolk | — |
|  | BFUKP050-12\|Simulium equinum\|COI-5P | United Kingdom, Norfolk | — |
|  | BFUKP051-12\|Simulium equinum\|COI-5P | United Kingdom, Norfolk | — |
|  | BFUKP025-12\|Simulium erythrocephalum\|COI-5P | United Kingdom, Norfolk | — |
|  | BFUKP026-12\|Simulium erythrocephalum\|COI-5P | United Kingdom, Norfolk | — |
|  | BFUKP027-12\|Simulium erythrocephalum\|COI-5P | United Kingdom, Norfolk | — |
|  | BSCHI728-17\|Tanypus kraatzi\|COI-5P | Sweden, Soedermanland | — |
|  | GBDP19214-15\|Tanypus kraatzi\|COI-5P\|LC050911 | Japan, Ibaraki, Tsukuba | — |
|  | BBDCM657-10\|Wiedemannia simplex\|COI-5P\|JF867352 | Canada, Ontario, Pukaskwa NP | — |
|  | BBDCM670-10\|Wiedemannia simplex\|COI-5P\|JF867364 | Canada, Ontario, Pukaskwa NP | — |
|  | BBDCN790-10\|Wiedemannia simplex\|COI-5P\|JF868288 | Canada, British Columbia, Kootenay NP | — |

**Part II**

**Results of the ABGD** **analysis for the combined dataset. Informative tables, graphs, distance matrices and histograms of the combined dataset and of each order, analyzed separately.**

Initial Partition with prior maximal distance P=5.99e-02
Distance JC69 Jukes-Cantor MinSlope=1.500000
**Group[ 0 ] n: 2 ;**id: XJDQD129-18|Amphinemura borealis XJDQD128-18|Amphinemura borealis
**Group[ 1 ] n: 43 ;**id: XJDQD130-18|Arcynopteryx compacta XJDQD131-18|Arcynopteryx compacta XJDQD132-18|Arcynopteryx compacta XJDQD133-18|Arcynopteryx compacta XJDQD134-18|Arcynopteryx compacta XJDQD135-18|Arcynopteryx compacta XJDQD136-18|Arcynopteryx compacta XJDQD137-18|Arcynopteryx compacta XJDQD138-18|Arcynopteryx compacta XJDQD139-18|Arcynopteryx compacta XJDQD140-18|Arcynopteryx compacta XJDQD141-18|Arcynopteryx compacta XJDQD142-18|Arcynopteryx compacta XJDQD143-18|Arcynopteryx compacta XJDQD144-18|Arcynopteryx compacta XJDQD145-18|Arcynopteryx compacta XJDQD146-18|Arcynopteryx compacta XJDQD147-18|Arcynopteryx compacta XJDQD148-18|Arcynopteryx compacta XJDQD149-18|Arcynopteryx compacta XJDQD150-18|Arcynopteryx compacta XJDQD151-18|Arcynopteryx compacta XJDQD152-18|Arcynopteryx compacta XJDQD153-18|Arcynopteryx compacta XJDQD154-18|Arcynopteryx compacta XJDQD155-18|Arcynopteryx compacta XJDQD156-18|Arcynopteryx compacta XJDQD157-18|Arcynopteryx compacta XJDQD158-18|Arcynopteryx compacta XJDQD159-18|Arcynopteryx compacta XJDQD160-18|Arcynopteryx compacta XJDQD161-18|Arcynopteryx compacta XJDQD162-18|Arcynopteryx compacta XJDQD163-18|Arcynopteryx compacta XJDQD164-18|Arcynopteryx compacta XJDQD165-18|Arcynopteryx compacta XJDQD166-18|Arcynopteryx compacta XJDQD167-18|Arcynopteryx compacta XJDQD168-18|Arcynopteryx compacta XJDQD169-18|Arcynopteryx compacta XJDQD170-18|Arcynopteryx compacta XJDQD171-18|Arcynopteryx compacta XJDQD172-18|Arcynopteryx compacta
**Group[ 2 ] n: 2 ;**id: XJDQD173-18|Capniidae XJDQD174-18|Capniidae
**Group[ 3 ] n: 17 ;**id: XJDQD175-18|Diura nanseni XJDQD176-18|Diura nanseni XJDQD177-18|Diura nanseni XJDQD178-18|Diura nanseni XJDQD179-18|Diura nanseni XJDQD180-18|Diura nanseni XJDQD181-18|Diura nanseni XJDQD182-18|Diura nanseni XJDQD183-18|Diura nanseni XJDQD184-18|Diura nanseni XJDQD185-18|Diura nanseni XJDQD186-18|Diura nanseni XJDQD187-18|Diura nanseni XJDQD188-18|Diura nanseni XJDQD189-18|Diura nanseni XJDQD190-18|Diura nanseni XJDQD191-18|Diura nanseni
**Group[ 4 ] n: 2 ;**id: XJDQD192-18|Haploperla ussurica XJDQD193-18|Haploperla ussurica
**Group[ 5 ] n: 2 ;**id: XJDQD194-18|Isoperla sp1 XJDQD195-18|Isoperla sp1
**Group[ 6 ] n: 6 ;**id: XJDQD196-18|Isoperla lunigera XJDQD197-18|Isoperla lunigera XJDQD198-18|Isoperla lunigera XJDQD199-18|Isoperla lunigera XJDQD200-18|Isoperla lunigera XJDQD201-18|Isoperla lunigera
**Group[ 7 ] n: 1 ;**id: XJDQD202-18|Nemoura cinerea
**Group[ 8 ] n: 5 ;**id: XJDQD203-18|Nemoura sp1 XJDQD204-18|Nemoura sp1 XJDQD205-18|Nemoura sp1 XJDQD206-18|Nemoura sp1 XJDQD207-18|Nemoura sp1
**Group[ 9 ] n: 22 ;**id: XJDQD208-18|Perlodes sp1 XJDQD209-18|Perlodes sp1 XJDQD210-18|Perlodes sp1 XJDQD211-18|Perlodes sp1 XJDQD212-18|Perlodes sp1 XJDQD213-18|Perlodes sp1 XJDQD214-18|Perlodes sp1 XJDQD215-18|Perlodes sp1 XJDQD216-18|Perlodes sp1 XJDQD217-18|Perlodes sp1 XJDQD218-18|Perlodes sp1 XJDQD219-18|Perlodes sp1 XJDQD220-18|Perlodes sp1 XJDQD221-18|Perlodes sp1 XJDQD222-18|Perlodes sp1 XJDQD223-18|Perlodes sp1 XJDQD224-18|Perlodes sp1 XJDQD225-18|Perlodes sp1 XJDQD226-18|Perlodes sp1 XJDQD227-18|Perlodes sp1 XJDQD228-18|Perlodes sp1 XJDQD229-18|Perlodes sp1
**Group[ 10 ] n: 4 ;**id: XJDQD230-18|Perlodes sp2 XJDQD231-18|Perlodes sp2 XJDQD232-18|Perlodes sp2 XJDQD233-18|Perlodes sp2
**Group[ 11 ] n: 6 ;**id: XJDQD234-18|Pteronarcys sp. XJ XJDQD235-18|Pteronarcys sp. XJ XJDQD236-18|Pteronarcys sp. XJ XJDQD237-18|Pteronarcys sp. XJ XJDQD238-18|Pteronarcys sp. XJ XJDQD239-18|Pteronarcys sp. XJ
**Group[ 12 ] n: 3 ;**id: XJDQD240-18|Taenionema sp. XJDQD241-18|Taenionema sp. XJDQD242-18|Taenionema sp.
**Group[ 13 ] n: 3 ;**id: XJDQD243-18|Agabus sp. XJDQD244-18|Agabus sp. XJDQD245-18|Agabus sp.
**Group[ 14 ] n: 3 ;**id: XJDQD246-18|Bembidion gratiosum XJDQD247-18|Bembidion gratiosum XJDQD248-18|Bembidion gratiosum
**Group[ 15 ] n: 1 ;**id: XJDQD249-18|Berosus frontifoveatus
**Group[ 16 ] n: 3 ;**id: XJDQD250-18|Colymbetes sp. XJDQD251-18|Colymbetes sp. XJDQD252-18|Colymbetes sp.
**Group[ 17 ] n: 1 ;**id: XJDQD253-18|Elmis sp.
**Group[ 18 ] n: 4 ;**id: XJDQD254-18|Graphoderus austriacus XJDQD255-18|Graphoderus austriacus XJDQD256-18|Graphoderus austriacus XJDQD257-18|Graphoderus austriacus
**Group[ 19 ] n: 3 ;**id: XJDQD258-18|Gyrinus marinus XJDQD259-18|Gyrinus marinus XJDQD260-18|Gyrinus marinus
**Group[ 20 ] n: 1 ;**id: XJDQD261-18|Gyrinus sp.
**Group[ 21 ] n: 2 ;**id: XJDQD262-18|Ilybius crassus XJDQD263-18|Ilybius crassus
**Group[ 22 ] n: 1 ;**id: XJDQD264-18|Laccophilus sp.
**Group[ 23 ] n: 1 ;**id: XJDQD265-18|Laccobius sp.
**Group[ 24 ] n: 11 ;**id: XJDQD266-18|Laccophilus minutus XJDQD267-18|Laccophilus minutus XJDQD268-18|Laccophilus minutus XJDQD269-18|Laccophilus minutus XJDQD270-18|Laccophilus minutus XJDQD271-18|Laccophilus minutus XJDQD272-18|Laccophilus minutus XJDQD273-18|Laccophilus minutus XJDQD274-18|Laccophilus minutus XJDQD275-18|Laccophilus minutus XJDQD276-18|Laccophilus minutus
**Group[ 25 ] n: 8 ;**id: XJDQD018-18|Branchiura sowerbyi XJDQD019-18|Branchiura sowerbyi XJDQD020-18|Branchiura sowerbyi XJDQD021-18|Branchiura sowerbyi XJDQD022-18|Branchiura sowerbyi XJDQD023-18|Branchiura sowerbyi XJDQD024-18|Branchiura sowerbyi XJDQD025-18|Branchiura sowerbyi
**Group[ 26 ] n: 1 ;**id: XJDQD026-18|Dendrodrilus rubidus
**Group[ 27 ] n: 1 ;**id: XJDQD027-18|Erpobdella sp2
**Group[ 28 ] n: 5 ;**id: XJDQD028-18|Erpobdella sp3 XJDQD029-18|Erpobdella sp3 XJDQD030-18|Erpobdella sp3 XJDQD031-18|Erpobdella sp3 XJDQD032-18|Erpobdella sp3
**Group[ 29 ] n: 33 ;**id: XJDQD033-18|Erpobdella sp1 XJDQD034-18|Erpobdella sp1 XJDQD035-18|Erpobdella sp1 XJDQD036-18|Erpobdella sp1 XJDQD037-18|Erpobdella sp1 XJDQD038-18|Erpobdella sp1 XJDQD039-18|Erpobdella sp1 XJDQD040-18|Erpobdella sp1 XJDQD041-18|Erpobdella sp1 XJDQD042-18|Erpobdella sp1 XJDQD043-18|Erpobdella sp1 XJDQD044-18|Erpobdella sp1 XJDQD045-18|Erpobdella sp1 XJDQD046-18|Erpobdella sp1 XJDQD047-18|Erpobdella sp1 XJDQD048-18|Erpobdella sp1 XJDQD049-18|Erpobdella sp1 XJDQD050-18|Erpobdella sp1 XJDQD051-18|Erpobdella sp1 XJDQD052-18|Erpobdella sp1 XJDQD053-18|Erpobdella sp1 XJDQD054-18|Erpobdella sp1 XJDQD055-18|Erpobdella sp1 XJDQD056-18|Erpobdella sp1 XJDQD057-18|Erpobdella sp1 XJDQD058-18|Erpobdella sp1 XJDQD059-18|Erpobdella sp1 XJDQD060-18|Erpobdella sp1 XJDQD061-18|Erpobdella sp1 XJDQD062-18|Erpobdella sp1 XJDQD063-18|Erpobdella sp1 XJDQD064-18|Erpobdella sp1 XJDQD065-18|Erpobdella sp1
**Group[ 30 ] n: 4 ;**id: XJDQD066-18|Glossosoma intermedium XJDQD067-18|Glossosoma intermedium XJDQD068-18|Glossosoma intermedium XJDQD069-18|Glossosoma intermedium
**Group[ 31 ] n: 6 ;**id: XJDQD070-18|Helobdella stagnalis XJDQD071-18|Helobdella stagnalis XJDQD072-18|Helobdella stagnalis XJDQD073-18|Helobdella stagnalis XJDQD074-18|Helobdella stagnalis XJDQD075-18|Helobdella stagnalis
**Group[ 32 ] n: 5 ;**id: XJDQD076-18|Limnodrilus hoffmeisteri XJDQD077-18|Limnodrilus hoffmeisteri XJDQD078-18|Limnodrilus hoffmeisteri XJDQD079-18|Limnodrilus hoffmeisteri XJDQD080-18|Limnodrilus hoffmeisteri
**Group[ 33 ] n: 1 ;**id: XJDQD081-18|Tubifex sp.
**Group[ 34 ] n: 5 ;**id: XJDQD082-18|Ischnura elegans XJDQD083-18|Ischnura elegans XJDQD084-18|Ischnura elegans XJDQD090-18|Ischnura elegans XJDQD091-18|Ischnura elegans
**Group[ 35 ] n: 1 ;**id: XJDQD085-18|Coenagrion sp.
**Group[ 36 ] n: 1 ;**id: XJDQD086-18|Enallagma cyathigerum
**Group[ 37 ] n: 3 ;**id: XJDQD087-18|Gomphus flavipes XJDQD088-18|Gomphus flavipes XJDQD089-18|Gomphus flavipes
**Group[ 38 ] n: 6 ;**id: XJDQD092-18|Ophiogomphus sp. XJDQD093-18|Ophiogomphus sp. XJDQD094-18|Ophiogomphus sp. XJDQD095-18|Ophiogomphus sp. XJDQD096-18|Ophiogomphus sp. XJDQD097-18|Ophiogomphus sp.
**Group[ 39 ] n: 7 ;**id: XJDQD098-18|Sympecma paedisca XJDQD099-18|Sympecma paedisca XJDQD100-18|Sympecma paedisca XJDQD101-18|Sympecma paedisca XJDQD102-18|Sympecma paedisca XJDQD103-18|Sympecma paedisca XJDQD104-18|Sympecma paedisca
**Group[ 40 ] n: 12 ;**id: XJDQD106-18|Agnetina brevipennis XJDQD107-18|Agnetina brevipennis XJDQD108-18|Agnetina brevipennis XJDQD109-18|Agnetina brevipennis XJDQD110-18|Agnetina brevipennis XJDQD111-18|Agnetina brevipennis XJDQD114-18|Agnetina brevipennis XJDQD123-18|Agnetina brevipennis XJDQD124-18|Agnetina brevipennis XJDQD125-18|Agnetina brevipennis XJDQD126-18|Agnetina brevipennis XJDQD127-18|Agnetina brevipennis
**Group[ 41 ] n: 10 ;**id: XJDQD112-18|Agnetina sp. XJDQD113-18|Agnetina sp. XJDQD115-18|Agnetina sp. XJDQD116-18|Agnetina sp. XJDQD117-18|Agnetina sp. XJDQD118-18|Agnetina sp. XJDQD119-18|Agnetina sp. XJDQD120-18|Agnetina sp. XJDQD121-18|Agnetina sp. XJDQD122-18|Agnetina sp.
**Group[ 42 ] n: 2 ;**id: XJDQD001-18|Gyraulus campressus XJDQD002-18|Gyraulus campressus
**Group[ 43 ] n: 2 ;**id: XJDQD003-18|Lymnaea stagnalis XJDQD004-18|Lymnaea stagnalis
**Group[ 44 ] n: 2 ;**id: XJDQD005-18|Physella acuta XJDQD006-18|Physella acuta
**Group[ 45 ] n: 8 ;**id: XJDQD007-18|Radix auricularia XJDQD008-18|Radix auricularia XJDQD009-18|Radix auricularia XJDQD010-18|Radix auricularia XJDQD011-18|Radix auricularia XJDQD012-18|Radix auricularia XJDQD013-18|Radix auricularia XJDQD014-18|Radix auricularia
**Group[ 46 ] n: 3 ;**id: XJDQD015-18|Radix zazurnensis XJDQD016-18|Radix zazurnensis XJDQD017-18|Radix zazurnensis
**Group[ 47 ] n: 2 ;**id: XJDQD277-18|Mecyclothorax sp. XJDQD278-18|Mecyclothorax sp.
**Group[ 48 ] n: 1 ;**id: XJDQD279-18|Nebrioporus sp.
**Group[ 49 ] n: 1 ;**id: XJDQD280-18|Noterus clavicornis
**Group[ 50 ] n: 2 ;**id: XJDQD281-18|Orectochilus sp. XJDQD282-18|Orectochilus sp.
**Group[ 51 ] n: 1 ;**id: XJDQD283-18|Philonthus sp.
**Group[ 52 ] n: 3 ;**id: XJDQD284-18|Rhantus notatus XJDQD285-18|Rhantus notatus XJDQD286-18|Rhantus notatus
**Group[ 53 ] n: 11 ;**id: XJDQD287-18|Rhantus suturalis XJDQD288-18|Rhantus suturalis XJDQD289-18|Rhantus suturalis XJDQD290-18|Rhantus suturalis XJDQD291-18|Rhantus suturalis XJDQD292-18|Rhantus suturalis XJDQD293-18|Rhantus suturalis XJDQD294-18|Rhantus suturalis XJDQD295-18|Rhantus suturalis XJDQD296-18|Rhantus suturalis XJDQD297-18|Rhantus suturalis
**Group[ 54 ] n: 1 ;**id: XJDQD298-18|Stenelmis sp.
**Group[ 55 ] n: 15 ;**id: XJDQD299-18|Arctopsyche amurensis XJDQD300-18|Arctopsyche amurensis XJDQD301-18|Arctopsyche amurensis XJDQD302-18|Arctopsyche amurensis XJDQD303-18|Arctopsyche amurensis XJDQD304-18|Arctopsyche amurensis XJDQD305-18|Arctopsyche amurensis XJDQD306-18|Arctopsyche amurensis XJDQD307-18|Arctopsyche amurensis XJDQD308-18|Arctopsyche amurensis XJDQD309-18|Arctopsyche amurensis XJDQD310-18|Arctopsyche amurensis XJDQD311-18|Arctopsyche amurensis XJDQD312-18|Arctopsyche amurensis XJDQD313-18|Arctopsyche amurensis
**Group[ 56 ] n: 10 ;**id: XJDQD314-18|Brachycentrus americanus XJDQD315-18|Brachycentrus americanus XJDQD316-18|Brachycentrus americanus XJDQD317-18|Brachycentrus americanus XJDQD318-18|Brachycentrus americanus XJDQD319-18|Brachycentrus americanus XJDQD320-18|Brachycentrus americanus XJDQD321-18|Brachycentrus americanus XJDQD322-18|Brachycentrus americanus XJDQD323-18|Brachycentrus americanus
**Group[ 57 ] n: 2 ;**id: XJDQD324-18|Dicosmoecus sp. XJDQD325-18|Dicosmoecus sp.
**Group[ 58 ] n: 2 ;**id: XJDQD326-18|Ecnomus tenellus XJDQD327-18|Ecnomus tenellus
**Group[ 59 ] n: 1 ;**id: XJDQD328-18|Glossosoma minutum
**Group[ 60 ] n: 3 ;**id: XJDQD329-18|Halesus tessellatus XJDQD330-18|Halesus tessellatus XJDQD331-18|Halesus tessellatus
**Group[ 61 ] n: 3 ;**id: XJDQD332-18|Hydropsyche sp. XJ XJDQD333-18|Hydropsyche sp. XJ XJDQD334-18|Hydropsyche sp. XJ
**Group[ 62 ] n: 2 ;**id: XJDQD335-18|Hydropsyche demavenda XJDQD336-18|Hydropsyche demavenda
**Group[ 63 ] n: 28 ;**id: XJDQD337-18|Hydropsyche kozhantschikovi XJDQD338-18|Hydropsyche kozhantschikovi XJDQD339-18|Hydropsyche kozhantschikovi XJDQD340-18|Hydropsyche kozhantschikovi XJDQD341-18|Hydropsyche kozhantschikovi XJDQD342-18|Hydropsyche kozhantschikovi XJDQD343-18|Hydropsyche kozhantschikovi XJDQD344-18|Hydropsyche kozhantschikovi XJDQD345-18|Hydropsyche kozhantschikovi XJDQD346-18|Hydropsyche kozhantschikovi XJDQD347-18|Hydropsyche kozhantschikovi XJDQD348-18|Hydropsyche kozhantschikovi XJDQD349-18|Hydropsyche kozhantschikovi XJDQD350-18|Hydropsyche kozhantschikovi XJDQD351-18|Hydropsyche kozhantschikovi XJDQD352-18|Hydropsyche kozhantschikovi XJDQD353-18|Hydropsyche kozhantschikovi XJDQD354-18|Hydropsyche kozhantschikovi XJDQD355-18|Hydropsyche kozhantschikovi XJDQD356-18|Hydropsyche kozhantschikovi XJDQD357-18|Hydropsyche kozhantschikovi XJDQD358-18|Hydropsyche kozhantschikovi XJDQD359-18|Hydropsyche kozhantschikovi XJDQD360-18|Hydropsyche kozhantschikovi XJDQD361-18|Hydropsyche kozhantschikovi XJDQD362-18|Hydropsyche kozhantschikovi XJDQD363-18|Hydropsyche kozhantschikovi XJDQD364-18|Hydropsyche kozhantschikovi
**Group[ 64 ] n: 1 ;**id: XJDQD365-18|Hydropsyche ornatula
**Group[ 65 ] n: 1 ;**id: XJDQD366-18|Hydropsyche pellucidula
**Group[ 66 ] n: 1 ;**id: XJDQD367-18|Hydropsyche valvata
**Group[ 67 ] n: 5 ;**id: XJDQD368-18|Potamyia czekanovskii XJDQD369-18|Potamyia czekanovskii XJDQD370-18|Potamyia czekanovskii XJDQD371-18|Potamyia czekanovskii XJDQD372-18|Potamyia czekanovskii
**Group[ 68 ] n: 1 ;**id: XJDQD373-18|Rhyacophila quadrifida
**Group[ 69 ] n: 7 ;**id: XJDQD374-18|Stenopsyche marmorata XJDQD375-18|Stenopsyche marmorata XJDQD376-18|Stenopsyche marmorata XJDQD377-18|Stenopsyche marmorata XJDQD378-18|Stenopsyche marmorata XJDQD379-18|Stenopsyche marmorata XJDQD380-18|Stenopsyche marmorata
**Group[ 70 ] n: 2 ;**id: XJDQD381-18|Callicorixa praeusta XJDQD382-18|Callicorixa praeusta
**Group[ 71 ] n: 2 ;**id: XJDQD383-18|Cheirochela sp. XJDQD384-18|Cheirochela sp.
**Group[ 72 ] n: 8 ;**id: XJDQD385-18|Corixa dentipes XJDQD386-18|Corixa dentipes XJDQD387-18|Corixa dentipes XJDQD388-18|Corixa dentipes XJDQD389-18|Corixa dentipes XJDQD390-18|Corixa dentipes XJDQD391-18|Corixa dentipes XJDQD392-18|Corixa dentipes
**Group[ 73 ] n: 1 ;**id: XJDQD393-18|Sigara falleni
**Group[ 74 ] n: 1 ;**id: XJDQD394-18|Hesperocorixa linnaei
**Group[ 75 ] n: 1 ;**id: XJDQD395-18|Ilyocoris cimicoides
**Group[ 76 ] n: 4 ;**id: XJDQD396-18|Nepa cinerea XJDQD397-18|Nepa cinerea XJDQD398-18|Nepa cinerea XJDQD399-18|Nepa cinerea
**Group[ 77 ] n: 25 ;**id: XJDQD400-18|Notonecta glauca XJDQD401-18|Notonecta glauca XJDQD402-18|Notonecta glauca XJDQD403-18|Notonecta glauca XJDQD404-18|Notonecta glauca XJDQD405-18|Notonecta glauca XJDQD406-18|Notonecta glauca XJDQD407-18|Notonecta glauca XJDQD408-18|Notonecta glauca XJDQD409-18|Notonecta glauca XJDQD410-18|Notonecta glauca XJDQD411-18|Notonecta glauca XJDQD412-18|Notonecta glauca XJDQD413-18|Notonecta glauca XJDQD414-18|Notonecta glauca XJDQD415-18|Notonecta glauca XJDQD416-18|Notonecta glauca XJDQD417-18|Notonecta glauca XJDQD418-18|Notonecta glauca XJDQD419-18|Notonecta glauca XJDQD420-18|Notonecta glauca XJDQD421-18|Notonecta glauca XJDQD422-18|Notonecta glauca XJDQD423-18|Notonecta glauca XJDQD424-18|Notonecta glauca
**Group[ 78 ] n: 4 ;**id: XJDQD425-18|Paracorixa concinna XJDQD426-18|Paracorixa concinna XJDQD427-18|Paracorixa concinna XJDQD428-18|Paracorixa concinna
**Group[ 79 ] n: 1 ;**id: XJDQD429-18|Ranatra linearis
**Group[ 80 ] n: 7 ;**id: XJDQD430-18|Sigara lateralis XJDQD431-18|Sigara lateralis XJDQD432-18|Sigara lateralis XJDQD433-18|Sigara lateralis XJDQD434-18|Sigara lateralis XJDQD435-18|Sigara lateralis XJDQD436-18|Sigara lateralis
**Group[ 81 ] n: 11 ;**id: XJDQD437-18|Sigara striata XJDQD438-18|Sigara striata XJDQD439-18|Sigara striata XJDQD440-18|Sigara striata XJDQD441-18|Sigara striata XJDQD442-18|Sigara striata XJDQD443-18|Sigara striata XJDQD444-18|Sigara striata XJDQD445-18|Sigara striata XJDQD446-18|Sigara striata XJDQD447-18|Sigara striata
**Group[ 82 ] n: 23 ;**id: XJDQD448-18|Ameletus montanus XJDQD449-18|Ameletus montanus XJDQD450-18|Ameletus montanus XJDQD451-18|Ameletus montanus XJDQD452-18|Ameletus montanus XJDQD453-18|Ameletus montanus XJDQD454-18|Ameletus montanus XJDQD455-18|Ameletus montanus XJDQD458-18|Ameletus montanus XJDQD459-18|Ameletus montanus XJDQD460-18|Ameletus montanus XJDQD461-18|Ameletus montanus XJDQD462-18|Ameletus montanus XJDQD463-18|Ameletus montanus XJDQD464-18|Ameletus montanus XJDQD467-18|Ameletus montanus XJDQD468-18|Ameletus montanus XJDQD469-18|Ameletus montanus XJDQD470-18|Ameletus montanus XJDQD471-18|Ameletus montanus XJDQD472-18|Ameletus montanus XJDQD473-18|Ameletus montanus XJDQD474-18|Ameletus montanus
**Group[ 83 ] n: 4 ;**id: XJDQD456-18|Ameletus montanus XJDQD457-18|Ameletus montanus XJDQD465-18|Ameletus montanus XJDQD466-18|Ameletus montanus
**Group[ 84 ] n: 26 ;**id: XJDQD475-18|Baetiella bispinosa XJDQD476-18|Baetiella bispinosa XJDQD477-18|Baetiella bispinosa XJDQD478-18|Baetiella bispinosa XJDQD479-18|Baetiella bispinosa XJDQD480-18|Baetiella bispinosa XJDQD481-18|Baetiella bispinosa XJDQD482-18|Baetiella bispinosa XJDQD483-18|Baetiella bispinosa XJDQD484-18|Baetiella bispinosa XJDQD485-18|Baetiella bispinosa XJDQD486-18|Baetiella bispinosa XJDQD487-18|Baetiella bispinosa XJDQD488-18|Baetiella bispinosa XJDQD489-18|Baetiella bispinosa XJDQD490-18|Baetiella bispinosa XJDQD491-18|Baetiella bispinosa XJDQD492-18|Baetiella bispinosa XJDQD493-18|Baetiella bispinosa XJDQD494-18|Baetiella bispinosa XJDQD495-18|Baetiella bispinosa XJDQD496-18|Baetiella bispinosa XJDQD497-18|Baetiella bispinosa XJDQD498-18|Baetiella bispinosa XJDQD499-18|Baetiella bispinosa XJDQD500-18|Baetiella bispinosa
**Group[ 85 ] n: 18 ;**id: XJDQD516-18|Baetiella sp1 XJDQD517-18|Baetiella sp1 XJDQD518-18|Baetiella sp1 XJDQD519-18|Baetiella sp1 XJDQD520-18|Baetiella sp1 XJDQD521-18|Baetiella sp1 XJDQD522-18|Baetiella sp1 XJDQD523-18|Baetiella sp1 XJDQD524-18|Baetiella sp1 XJDQD525-18|Baetiella sp1 XJDQD526-18|Baetiella sp1 XJDQD527-18|Baetiella sp1 XJDQD528-18|Baetiella sp1 XJDQD529-18|Baetiella sp1 XJDQD530-18|Baetiella sp1 XJDQD531-18|Baetiella sp1 XJDQD532-18|Baetiella sp1 XJDQD533-18|Baetiella sp1
**Group[ 86 ] n: 9 ;**id: XJDQD534-18|Baetiella sp2 XJDQD535-18|Baetiella sp2 XJDQD536-18|Baetiella sp2 XJDQD537-18|Baetiella sp2 XJDQD538-18|Baetiella sp2 XJDQD539-18|Baetiella sp2 XJDQD540-18|Baetiella sp2 XJDQD541-18|Baetiella sp2 XJDQD542-18|Baetiella sp2
**Group[ 87 ] n: 23 ;**id: XJDQD543-18|Baetis braaschi XJDQD544-18|Baetis braaschi XJDQD545-18|Baetis braaschi XJDQD546-18|Baetis braaschi XJDQD547-18|Baetis braaschi XJDQD548-18|Baetis braaschi XJDQD549-18|Baetis braaschi XJDQD550-18|Baetis braaschi XJDQD551-18|Baetis braaschi XJDQD552-18|Baetis braaschi XJDQD553-18|Baetis braaschi XJDQD554-18|Baetis braaschi XJDQD555-18|Baetis braaschi XJDQD556-18|Baetis braaschi XJDQD557-18|Baetis braaschi XJDQD558-18|Baetis braaschi XJDQD559-18|Baetis braaschi XJDQD560-18|Baetis braaschi XJDQD561-18|Baetis braaschi XJDQD562-18|Baetis braaschi XJDQD563-18|Baetis braaschi XJDQD564-18|Baetis braaschi XJDQD565-18|Baetis braaschi
**Group[ 88 ] n: 10 ;**id: XJDQD566-18|Baetis phoebus XJDQD567-18|Baetis phoebus XJDQD568-18|Baetis phoebus XJDQD569-18|Baetis phoebus XJDQD570-18|Baetis phoebus XJDQD571-18|Baetis phoebus XJDQD572-18|Baetis phoebus XJDQD573-18|Baetis phoebus XJDQD574-18|Baetis phoebus XJDQD575-18|Baetis phoebus
**Group[ 89 ] n: 9 ;**id: XJDQD576-18|Baetis sp1 XJDQD577-18|Baetis sp1 XJDQD578-18|Baetis sp1 XJDQD579-18|Baetis sp1 XJDQD580-18|Baetis sp1 XJDQD581-18|Baetis sp1 XJDQD582-18|Baetis sp1 XJDQD583-18|Baetis sp1 XJDQD584-18|Baetis sp1
**Group[ 90 ] n: 1 ;**id: XJDQD585-18|Caenis lactea
**Group[ 91 ] n: 7 ;**id: XJDQD586-18|Caenis robusta XJDQD587-18|Caenis robusta XJDQD588-18|Caenis robusta XJDQD589-18|Caenis robusta XJDQD590-18|Caenis robusta XJDQD591-18|Caenis robusta XJDQD592-18|Caenis robusta
**Group[ 92 ] n: 1 ;**id: XJDQD593-18|Caenis sp1
**Group[ 93 ] n: 1 ;**id: XJDQD594-18|Caenis sp2
**Group[ 94 ] n: 3 ;**id: XJDQD595-18|Choroterpes facialis XJDQD596-18|Choroterpes facialis XJDQD597-18|Choroterpes facialis
**Group[ 95 ] n: 18 ;**id: XJDQD598-18|Cinygmula sp1 XJDQD599-18|Cinygmula sp1 XJDQD600-18|Cinygmula sp1 XJDQD601-18|Cinygmula sp1 XJDQD602-18|Cinygmula sp1 XJDQD603-18|Cinygmula sp1 XJDQD604-18|Cinygmula sp1 XJDQD605-18|Cinygmula sp1 XJDQD606-18|Cinygmula sp1 XJDQD607-18|Cinygmula sp1 XJDQD608-18|Cinygmula sp1 XJDQD609-18|Cinygmula sp1 XJDQD610-18|Cinygmula sp1 XJDQD611-18|Cinygmula sp1 XJDQD612-18|Cinygmula sp1 XJDQD613-18|Cinygmula sp1 XJDQD614-18|Cinygmula sp1 XJDQD615-18|Cinygmula sp1
**Group[ 96 ] n: 10 ;**id: XJDQD616-18|Cinygmula sp2 XJDQD617-18|Cinygmula sp2 XJDQD618-18|Cinygmula sp2 XJDQD619-18|Cinygmula sp2 XJDQD620-18|Cinygmula sp2 XJDQD621-18|Cinygmula sp2 XJDQD622-18|Cinygmula sp2 XJDQD623-18|Cinygmula sp2 XJDQD624-18|Cinygmula sp2 XJDQD625-18|Cinygmula sp2
**Group[ 97 ] n: 3 ;**id: XJDQD626-18|Cloeon dipterum XJDQD627-18|Cloeon dipterum XJDQD628-18|Cloeon dipterum
**Group[ 98 ] n: 9 ;**id: XJDQD629-18|Drunella tricantha XJDQD630-18|Drunella tricantha XJDQD631-18|Drunella tricantha XJDQD632-18|Drunella tricantha XJDQD633-18|Drunella tricantha XJDQD634-18|Drunella tricantha XJDQD635-18|Drunella tricantha XJDQD636-18|Drunella tricantha XJDQD637-18|Drunella tricantha
**Group[ 99 ] n: 7 ;**id: XJDQD638-18|Drunella fusongensis XJDQD639-18|Drunella fusongensis XJDQD640-18|Drunella fusongensis XJDQD641-18|Drunella fusongensis XJDQD642-18|Drunella fusongensis XJDQD643-18|Drunella fusongensis XJDQD644-18|Drunella fusongensis
**Group[ 100 ] n: 19 ;**id: XJDQD645-18|Ecdyonurus kibunensis XJDQD646-18|Ecdyonurus kibunensis XJDQD647-18|Ecdyonurus kibunensis XJDQD648-18|Ecdyonurus kibunensis XJDQD649-18|Ecdyonurus kibunensis XJDQD650-18|Ecdyonurus kibunensis XJDQD651-18|Ecdyonurus kibunensis XJDQD652-18|Ecdyonurus kibunensis XJDQD653-18|Ecdyonurus kibunensis XJDQD654-18|Ecdyonurus kibunensis XJDQD655-18|Ecdyonurus kibunensis XJDQD656-18|Ecdyonurus kibunensis XJDQD657-18|Ecdyonurus kibunensis XJDQD658-18|Ecdyonurus kibunensis XJDQD659-18|Ecdyonurus kibunensis XJDQD660-18|Ecdyonurus kibunensis XJDQD661-18|Ecdyonurus kibunensis XJDQD662-18|Ecdyonurus kibunensis XJDQD663-18|Ecdyonurus kibunensis
**Group[ 101 ] n: 5 ;**id: XJDQD682-18|Ecdyonurus sp. XJDQD683-18|Ecdyonurus sp. XJDQD684-18|Ecdyonurus sp. XJDQD685-18|Ecdyonurus sp. XJDQD686-18|Ecdyonurus sp.
**Group[ 102 ] n: 38 ;**id: XJDQD687-18|Epeorus sp1 XJDQD688-18|Epeorus sp1 XJDQD689-18|Epeorus sp1 XJDQD690-18|Epeorus sp1 XJDQD691-18|Epeorus sp1 XJDQD692-18|Epeorus sp1 XJDQD693-18|Epeorus sp1 XJDQD694-18|Epeorus sp1 XJDQD695-18|Epeorus sp1 XJDQD696-18|Epeorus sp1 XJDQD697-18|Epeorus sp1 XJDQD698-18|Epeorus sp1 XJDQD699-18|Epeorus sp1 XJDQD700-18|Epeorus sp1 XJDQD701-18|Epeorus sp1 XJDQD702-18|Epeorus sp1 XJDQD703-18|Epeorus sp1 XJDQD704-18|Epeorus sp1 XJDQD705-18|Epeorus sp1 XJDQD706-18|Epeorus sp1 XJDQD707-18|Epeorus sp1 XJDQD708-18|Epeorus sp1 XJDQD709-18|Epeorus sp1 XJDQD710-18|Epeorus sp1 XJDQD711-18|Epeorus sp1 XJDQD712-18|Epeorus sp1 XJDQD713-18|Epeorus sp1 XJDQD714-18|Epeorus sp1 XJDQD715-18|Epeorus sp1 XJDQD716-18|Epeorus sp1 XJDQD717-18|Epeorus sp1 XJDQD718-18|Epeorus sp1 XJDQD719-18|Epeorus sp1 XJDQD720-18|Epeorus sp1 XJDQD721-18|Epeorus sp1 XJDQD722-18|Epeorus sp1 XJDQD723-18|Epeorus sp1 XJDQD724-18|Epeorus sp1
**Group[ 103 ] n: 32 ;**id: XJDQD725-18|Epeorus sp2 XJDQD726-18|Epeorus sp2 XJDQD727-18|Epeorus sp2 XJDQD728-18|Epeorus sp2 XJDQD729-18|Epeorus sp2 XJDQD730-18|Epeorus sp2 XJDQD731-18|Epeorus sp2 XJDQD732-18|Epeorus sp2 XJDQD733-18|Epeorus sp2 XJDQD734-18|Epeorus sp2 XJDQD735-18|Epeorus sp2 XJDQD736-18|Epeorus sp2 XJDQD737-18|Epeorus sp2 XJDQD738-18|Epeorus sp2 XJDQD739-18|Epeorus sp2 XJDQD740-18|Epeorus sp2 XJDQD741-18|Epeorus sp2 XJDQD742-18|Epeorus sp2 XJDQD743-18|Epeorus sp2 XJDQD744-18|Epeorus sp2 XJDQD745-18|Epeorus sp2 XJDQD746-18|Epeorus sp2 XJDQD747-18|Epeorus sp2 XJDQD748-18|Epeorus sp2 XJDQD749-18|Epeorus sp2 XJDQD750-18|Epeorus sp2 XJDQD751-18|Epeorus sp2 XJDQD752-18|Epeorus sp2 XJDQD753-18|Epeorus sp2 XJDQD754-18|Epeorus sp2 XJDQD755-18|Epeorus sp2 XJDQD756-18|Epeorus sp2
**Group[ 104 ] n: 14 ;**id: XJDQD757-18|Epeorus sp3 XJDQD758-18|Epeorus sp3 XJDQD759-18|Epeorus sp3 XJDQD760-18|Epeorus sp3 XJDQD761-18|Epeorus sp3 XJDQD762-18|Epeorus sp3 XJDQD763-18|Epeorus sp3 XJDQD764-18|Epeorus sp3 XJDQD765-18|Epeorus sp3 XJDQD766-18|Epeorus sp3 XJDQD767-18|Epeorus sp3 XJDQD768-18|Epeorus sp3 XJDQD769-18|Epeorus sp3 XJDQD770-18|Epeorus sp3
**Group[ 105 ] n: 6 ;**id: XJDQD771-18|Epeorus sp4 XJDQD772-18|Epeorus sp4 XJDQD773-18|Epeorus sp4 XJDQD774-18|Epeorus sp4 XJDQD775-18|Epeorus sp4 XJDQD776-18|Epeorus sp4
**Group[ 106 ] n: 6 ;**id: XJDQD777-18|Epeorus sp5 XJDQD778-18|Epeorus sp5 XJDQD779-18|Epeorus sp5 XJDQD780-18|Epeorus sp5 XJDQD781-18|Epeorus sp5 XJDQD782-18|Epeorus sp5
**Group[ 107 ] n: 2 ;**id: XJDQD783-18|Ephemera supposita XJDQD784-18|Ephemera supposita
**Group[ 108 ] n: 5 ;**id: XJDQD785-18|Ephemerella aurivillii XJDQD786-18|Ephemerella aurivillii XJDQD787-18|Ephemerella aurivillii XJDQD788-18|Ephemerella aurivillii XJDQD789-18|Ephemerella aurivillii
**Group[ 109 ] n: 5 ;**id: XJDQD790-18|Ephemerella mucronata XJDQD791-18|Ephemerella mucronata XJDQD792-18|Ephemerella mucronata XJDQD793-18|Ephemerella mucronata XJDQD1258-18|Ephemerella mucronata
**Group[ 110 ] n: 5 ;**id: XJDQD794-18|Heptagenia dalecarlica XJDQD795-18|Heptagenia dalecarlica XJDQD796-18|Heptagenia dalecarlica XJDQD797-18|Heptagenia dalecarlica XJDQD1259-18|Heptagenia dalecarlica
**Group[ 111 ] n: 10 ;**id: XJDQD798-18|Heptagenia flava XJDQD799-18|Heptagenia flava XJDQD800-18|Heptagenia flava XJDQD801-18|Heptagenia flava XJDQD802-18|Heptagenia flava XJDQD803-18|Heptagenia flava XJDQD804-18|Heptagenia flava XJDQD805-18|Heptagenia flava XJDQD806-18|Heptagenia flava XJDQD807-18|Heptagenia flava
**Group[ 112 ] n: 6 ;**id: XJDQD808-18|Heptagenia sp1 XJDQD809-18|Heptagenia sp1 XJDQD810-18|Heptagenia sp1 XJDQD811-18|Heptagenia sp1 XJDQD812-18|Heptagenia sp1 XJDQD813-18|Heptagenia sp1
**Group[ 113 ] n: 8 ;**id: XJDQD814-18|Iron maculates XJDQD815-18|Iron maculates XJDQD816-18|Iron maculates XJDQD817-18|Iron maculates XJDQD818-18|Iron maculates XJDQD819-18|Iron maculates XJDQD820-18|Iron maculates XJDQD821-18|Iron maculates
**Group[ 114 ] n: 34 ;**id: XJDQD822-18|Iron pellucidus XJDQD823-18|Iron pellucidus XJDQD824-18|Iron pellucidus XJDQD825-18|Iron pellucidus XJDQD826-18|Iron pellucidus XJDQD827-18|Iron pellucidus XJDQD828-18|Iron pellucidus XJDQD829-18|Iron pellucidus XJDQD830-18|Iron pellucidus XJDQD831-18|Iron pellucidus XJDQD832-18|Iron pellucidus XJDQD833-18|Iron pellucidus XJDQD834-18|Iron pellucidus XJDQD835-18|Iron pellucidus XJDQD836-18|Iron pellucidus XJDQD837-18|Iron pellucidus XJDQD838-18|Iron pellucidus XJDQD839-18|Iron pellucidus XJDQD840-18|Iron pellucidus XJDQD841-18|Iron pellucidus XJDQD842-18|Iron pellucidus XJDQD843-18|Iron pellucidus XJDQD844-18|Iron pellucidus XJDQD845-18|Iron pellucidus XJDQD846-18|Iron pellucidus XJDQD847-18|Iron pellucidus XJDQD848-18|Iron pellucidus XJDQD849-18|Iron pellucidus XJDQD850-18|Iron pellucidus XJDQD851-18|Iron pellucidus XJDQD852-18|Iron pellucidus XJDQD853-18|Iron pellucidus XJDQD854-18|Iron pellucidus XJDQD855-18|Iron pellucidus
**Group[ 115 ] n: 5 ;**id: XJDQD856-18|Oligoneuriella sp1 XJDQD857-18|Oligoneuriella sp1 XJDQD858-18|Oligoneuriella sp1 XJDQD859-18|Oligoneuriella sp1 XJDQD860-18|Oligoneuriella sp1
**Group[ 116 ] n: 18 ;**id: XJDQD861-18|Penelomax sp1 XJDQD862-18|Penelomax sp1 XJDQD863-18|Penelomax sp1 XJDQD864-18|Penelomax sp1 XJDQD865-18|Penelomax sp1 XJDQD866-18|Penelomax sp1 XJDQD867-18|Penelomax sp1 XJDQD868-18|Penelomax sp1 XJDQD869-18|Penelomax sp1 XJDQD870-18|Penelomax sp1 XJDQD871-18|Penelomax sp1 XJDQD872-18|Penelomax sp1 XJDQD873-18|Penelomax sp1 XJDQD874-18|Penelomax sp1 XJDQD875-18|Penelomax sp1 XJDQD876-18|Penelomax sp1 XJDQD877-18|Penelomax sp1 XJDQD878-18|Penelomax sp1
**Group[ 117 ] n: 21 ;**id: XJDQD879-18|Potamanthus tongitibius XJDQD880-18|Potamanthus tongitibius XJDQD881-18|Potamanthus tongitibius XJDQD882-18|Potamanthus tongitibius XJDQD883-18|Potamanthus tongitibius XJDQD884-18|Potamanthus tongitibius XJDQD885-18|Potamanthus tongitibius XJDQD886-18|Potamanthus tongitibius XJDQD887-18|Potamanthus tongitibius XJDQD888-18|Potamanthus tongitibius XJDQD889-18|Potamanthus tongitibius XJDQD890-18|Potamanthus tongitibius XJDQD891-18|Potamanthus tongitibius XJDQD892-18|Potamanthus tongitibius XJDQD893-18|Potamanthus tongitibius XJDQD894-18|Potamanthus tongitibius XJDQD895-18|Potamanthus tongitibius XJDQD896-18|Potamanthus tongitibius XJDQD897-18|Potamanthus tongitibius XJDQD898-18|Potamanthus tongitibius XJDQD899-18|Potamanthus tongitibius
**Group[ 118 ] n: 6 ;**id: XJDQD900-18|Rhithrogena lepnevae XJDQD901-18|Rhithrogena lepnevae XJDQD902-18|Rhithrogena lepnevae XJDQD903-18|Rhithrogena lepnevae XJDQD904-18|Rhithrogena lepnevae XJDQD905-18|Rhithrogena lepnevae
**Group[ 119 ] n: 14 ;**id: XJDQD906-18|Rhithrogena tianshanica XJDQD907-18|Rhithrogena tianshanica XJDQD908-18|Rhithrogena tianshanica XJDQD909-18|Rhithrogena tianshanica XJDQD910-18|Rhithrogena tianshanica XJDQD911-18|Rhithrogena tianshanica XJDQD912-18|Rhithrogena tianshanica XJDQD913-18|Rhithrogena tianshanica XJDQD914-18|Rhithrogena tianshanica XJDQD915-18|Rhithrogena tianshanica XJDQD916-18|Rhithrogena tianshanica XJDQD917-18|Rhithrogena tianshanica XJDQD918-18|Rhithrogena tianshanica XJDQD919-18|Rhithrogena tianshanica
**Group[ 120 ] n: 47 ;**id: XJDQD920-18|Serratella ignita XJDQD921-18|Serratella ignita XJDQD922-18|Serratella ignita XJDQD923-18|Serratella ignita XJDQD924-18|Serratella ignita XJDQD925-18|Serratella ignita XJDQD926-18|Serratella ignita XJDQD927-18|Serratella ignita XJDQD928-18|Serratella ignita XJDQD929-18|Serratella ignita XJDQD930-18|Serratella ignita XJDQD931-18|Serratella ignita XJDQD932-18|Serratella ignita XJDQD933-18|Serratella ignita XJDQD934-18|Serratella ignita XJDQD935-18|Serratella ignita XJDQD936-18|Serratella ignita XJDQD937-18|Serratella ignita XJDQD938-18|Serratella ignita XJDQD939-18|Serratella ignita XJDQD940-18|Serratella ignita XJDQD941-18|Serratella ignita XJDQD942-18|Serratella ignita XJDQD943-18|Serratella ignita XJDQD944-18|Serratella ignita XJDQD945-18|Serratella ignita XJDQD946-18|Serratella ignita XJDQD947-18|Serratella ignita XJDQD948-18|Serratella ignita XJDQD949-18|Serratella ignita XJDQD950-18|Serratella ignita XJDQD951-18|Serratella ignita XJDQD952-18|Serratella ignita XJDQD953-18|Serratella ignita XJDQD954-18|Serratella ignita XJDQD955-18|Serratella ignita XJDQD956-18|Serratella ignita XJDQD957-18|Serratella ignita XJDQD958-18|Serratella ignita XJDQD959-18|Serratella ignita XJDQD960-18|Serratella ignita XJDQD961-18|Serratella ignita XJDQD962-18|Serratella ignita XJDQD963-18|Serratella ignita XJDQD964-18|Serratella ignita XJDQD965-18|Serratella ignita XJDQD966-18|Serratella ignita
**Group[ 121 ] n: 1 ;**id: XJDQD967-18|Siphlonurus sp.
**Group[ 122 ] n: 12 ;**id: XJDQD968-18|Uracanthella rufa XJDQD969-18|Uracanthella rufa XJDQD970-18|Uracanthella rufa XJDQD971-18|Uracanthella rufa XJDQD972-18|Uracanthella rufa XJDQD973-18|Uracanthella rufa XJDQD974-18|Uracanthella rufa XJDQD975-18|Uracanthella rufa XJDQD976-18|Uracanthella rufa XJDQD977-18|Uracanthella rufa XJDQD978-18|Uracanthella rufa XJDQD979-18|Uracanthella rufa
**Group[ 123 ] n: 1 ;**id: XJDQD980-18|Ablabesmyia sp. XJ
**Group[ 124 ] n: 5 ;**id: XJDQD981-18|Anopheles darlingi XJDQD982-18|Anopheles darlingi XJDQD983-18|Anopheles darlingi XJDQD984-18|Anopheles darlingi XJDQD985-18|Anopheles darlingi
**Group[ 125 ] n: 6 ;**id: XJDQD986-18|Atherix sp. XJ XJDQD987-18|Atherix sp. XJ XJDQD988-18|Atherix sp. XJ XJDQD989-18|Atherix sp. XJ XJDQD990-18|Atherix sp. XJ XJDQD991-18|Atherix sp. XJ
**Group[ 126 ] n: 5 ;**id: XJDQD992-18|Blephariceridae XJDQD993-18|Blephariceridae XJDQD994-18|Blephariceridae XJDQD995-18|Blephariceridae XJDQD996-18|Blephariceridae
**Group[ 127 ] n: 3 ;**id: XJDQD997-18|Chaoborus flavicans XJDQD998-18|Chaoborus flavicans XJDQD999-18|Chaoborus flavicans
**Group[ 128 ] n: 1 ;**id: XJDQD1000-18|Chironomus alpestris
**Group[ 129 ] n: 26 ;**id: XJDQD1001-18|Chironomus annularius XJDQD1002-18|Chironomus annularius XJDQD1003-18|Chironomus annularius XJDQD1004-18|Chironomus annularius XJDQD1005-18|Chironomus annularius XJDQD1006-18|Chironomus annularius XJDQD1007-18|Chironomus annularius XJDQD1008-18|Chironomus annularius XJDQD1009-18|Chironomus annularius XJDQD1010-18|Chironomus annularius XJDQD1011-18|Chironomus annularius XJDQD1012-18|Chironomus annularius XJDQD1013-18|Chironomus annularius XJDQD1014-18|Chironomus annularius XJDQD1015-18|Chironomus annularius XJDQD1016-18|Chironomus annularius XJDQD1017-18|Chironomus annularius XJDQD1018-18|Chironomus annularius XJDQD1019-18|Chironomus annularius XJDQD1020-18|Chironomus annularius XJDQD1021-18|Chironomus annularius XJDQD1022-18|Chironomus annularius XJDQD1023-18|Chironomus annularius XJDQD1024-18|Chironomus annularius XJDQD1025-18|Chironomus annularius XJDQD1026-18|Chironomus annularius
**Group[ 130 ] n: 1 ;**id: XJDQD1027-18|Chironomus anthracinus
**Group[ 131 ] n: 8 ;**id: XJDQD1028-18|Chironomus pallidivittatus XJDQD1029-18|Chironomus pallidivittatus XJDQD1030-18|Chironomus pallidivittatus XJDQD1031-18|Chironomus pallidivittatus XJDQD1032-18|Chironomus pallidivittatus XJDQD1033-18|Chironomus pallidivittatus XJDQD1034-18|Chironomus pallidivittatus XJDQD1035-18|Chironomus pallidivittatus
**Group[ 132 ] n: 4 ;**id: XJDQD1036-18|Chironomus plumosus XJDQD1037-18|Chironomus plumosus XJDQD1038-18|Chironomus plumosus XJDQD1039-18|Chironomus plumosus
**Group[ 133 ] n: 2 ;**id: XJDQD1040-18|Chironemus sp. XJDQD1117-18|Chironomus heterodentatus
**Group[ 134 ] n: 3 ;**id: XJDQD1041-18|Cricotopus bicinctus XJDQD1042-18|Cricotopus bicinctus XJDQD1043-18|Cricotopus bicinctus
**Group[ 135 ] n: 4 ;**id: XJDQD1044-18|Cricotopus ornatus XJDQD1045-18|Cricotopus ornatus XJDQD1046-18|Cricotopus ornatus XJDQD1047-18|Cricotopus ornatus
**Group[ 136 ] n: 1 ;**id: XJDQD1048-18|Cricotopus sp. XJA
**Group[ 137 ] n: 3 ;**id: XJDQD1049-18|Cricotopus sp. XJB XJDQD1050-18|Cricotopus sp. XJB XJDQD1051-18|Cricotopus sp. XJB
**Group[ 138 ] n: 2 ;**id: XJDQD1052-18|Cricotopus sylvestris XJDQD1053-18|Cricotopus sylvestris
**Group[ 139 ] n: 2 ;**id: XJDQD1054-18|Cricotopus triannulatus XJDQD1055-18|Cricotopus triannulatus
**Group[ 140 ] n: 6 ;**id: XJDQD1056-18|Cricotopus trifascia XJDQD1057-18|Cricotopus trifascia XJDQD1058-18|Cricotopus trifascia XJDQD1059-18|Cricotopus trifascia XJDQD1060-18|Cricotopus trifascia XJDQD1061-18|Cricotopus trifascia
**Group[ 141 ] n: 1 ;**id: XJDQD1062-18|Cryptochironomus supplicans
**Group[ 142 ] n: 1 ;**id: XJDQD1063-18|Culicoides sp. XJ
**Group[ 143 ] n: 1 ;**id: XJDQD1064-18|Diamesa sp. XJA
**Group[ 144 ] n: 13 ;**id: XJDQD1065-18|Diamesa sp. XJB XJDQD1066-18|Diamesa sp. XJB XJDQD1067-18|Diamesa sp. XJB XJDQD1068-18|Diamesa sp. XJB XJDQD1069-18|Diamesa sp. XJB XJDQD1070-18|Diamesa sp. XJB XJDQD1071-18|Diamesa sp. XJB XJDQD1072-18|Diamesa sp. XJB XJDQD1073-18|Diamesa sp. XJB XJDQD1074-18|Diamesa sp. XJC XJDQD1075-18|Diamesa sp. XJC XJDQD1076-18|Diamesa sp. XJC XJDQD1077-18|Diamesa sp. XJC
**Group[ 145 ] n: 2 ;**id: XJDQD1078-18|Diamesa tonsa XJDQD1079-18|Diamesa tonsa
**Group[ 146 ] n: 6 ;**id: XJDQD1080-18|Dicranota guerini XJDQD1081-18|Dicranota guerini XJDQD1082-18|Dicranota guerini XJDQD1084-18|Dicranota guerini XJDQD1085-18|Dicranota guerini XJDQD1086-18|Dicranota guerini
**Group[ 147 ] n: 1 ;**id: XJDQD1083-18|Dicranota guerini
**Group[ 148 ] n: 2 ;**id: XJDQD1087-18|Endochironomus tendens XJDQD1088-18|Endochironomus tendens
**Group[ 149 ] n: 2 ;**id: XJDQD1089-18|Eukiefferiella sp. XJ XJDQD1090-18|Eukiefferiella sp. XJ
**Group[ 150 ] n: 13 ;**id: XJDQD1091-18|Euryhapsis sp. XJDQD1092-18|Euryhapsis sp. XJDQD1093-18|Euryhapsis sp. XJDQD1094-18|Euryhapsis sp. XJDQD1095-18|Euryhapsis sp. XJDQD1096-18|Euryhapsis sp. XJDQD1097-18|Euryhapsis sp. XJDQD1098-18|Euryhapsis sp. XJDQD1099-18|Euryhapsis sp. XJDQD1100-18|Euryhapsis sp. XJDQD1101-18|Euryhapsis sp. XJDQD1102-18|Euryhapsis sp. XJDQD1103-18|Euryhapsis sp.
**Group[ 151 ] n: 6 ;**id: XJDQD1104-18|Geranomyia sp. XJ XJDQD1105-18|Geranomyia sp. XJ XJDQD1106-18|Geranomyia sp. XJ XJDQD1107-18|Geranomyia sp. XJ XJDQD1108-18|Geranomyia sp. XJ XJDQD1109-18|Geranomyia sp. XJ
**Group[ 152 ] n: 6 ;**id: XJDQD1110-18|Glyptotendipes sp. XJ XJDQD1111-18|Glyptotendipes sp. XJ XJDQD1112-18|Glyptotendipes sp. XJ XJDQD1113-18|Glyptotendipes sp. XJ XJDQD1114-18|Glyptotendipes sp. XJ XJDQD1115-18|Glyptotendipes sp. XJ
**Group[ 153 ] n: 1 ;**id: XJDQD1116-18|Harnischia curtilamellata
**Group[ 154 ] n: 5 ;**id: XJDQD1118-18|Hexatoma sp. XJ XJDQD1119-18|Hexatoma sp. XJ XJDQD1120-18|Hexatoma sp. XJ XJDQD1121-18|Hexatoma sp. XJ XJDQD1122-18|Hexatoma sp. XJ
**Group[ 155 ] n: 1 ;**id: XJDQD1123-18|Limonia sp.
**Group[ 156 ] n: 1 ;**id: XJDQD1124-18|Lipiniella sp.
**Group[ 157 ] n: 2 ;**id: XJDQD1125-18|Macropelopia johnseni XJDQD1126-18|Macropelopia johnseni
**Group[ 158 ] n: 1 ;**id: XJDQD1127-18|Monodiamesa sp.
**Group[ 159 ] n: 1 ;**id: XJDQD1128-18|Nephrotoma sp.
**Group[ 160 ] n: 2 ;**id: XJDQD1129-18|Orthocladius nitidoscutellatus XJDQD1130-18|Orthocladius nitidoscutellatus
**Group[ 161 ] n: 1 ;**id: XJDQD1131-18|Orthocladius saxicola
**Group[ 162 ] n: 6 ;**id: XJDQD1132-18|Orthocladius sp. XJA XJDQD1133-18|Orthocladius sp. XJA XJDQD1134-18|Orthocladius sp. XJA XJDQD1135-18|Orthocladius sp. XJA XJDQD1136-18|Orthocladius sp. XJA XJDQD1137-18|Orthocladius sp. XJA
**Group[ 163 ] n: 9 ;**id: XJDQD1138-18|Orthocladius sp. XJB XJDQD1139-18|Orthocladius sp. XJB XJDQD1140-18|Orthocladius sp. XJB XJDQD1141-18|Orthocladius sp. XJB XJDQD1142-18|Orthocladius sp. XJB XJDQD1143-18|Orthocladius sp. XJB XJDQD1144-18|Orthocladius sp. XJB XJDQD1145-18|Orthocladius sp. XJB XJDQD1146-18|Orthocladius sp. XJB
**Group[ 164 ] n: 11 ;**id: XJDQD1147-18|Orthocladius thienemanni XJDQD1148-18|Orthocladius thienemanni XJDQD1149-18|Orthocladius thienemanni XJDQD1150-18|Orthocladius thienemanni XJDQD1151-18|Orthocladius thienemanni XJDQD1152-18|Orthocladius thienemanni XJDQD1153-18|Orthocladius thienemanni XJDQD1272-18|Orthocladius thienemanni XJDQD1273-18|Orthocladius thienemanni XJDQD1274-18|Orthocladius thienemanni XJDQD1275-18|Orthocladius thienemanni
**Group[ 165 ] n: 1 ;**id: XJDQD1154-18|Pagastia sp. XJA
**Group[ 166 ] n: 2 ;**id: XJDQD1168-18|Phaenopsectra sp1 XJDQD1169-18|Phaenopsectra sp1
**Group[ 167 ] n: 6 ;**id: XJDQD1170-18|Polypedilum bullum XJDQD1173-18|Polypedilum laetum XJDQD1174-18|Polypedilum laetum XJDQD1175-18|Polypedilum laetum XJDQD1176-18|Polypedilum laetum XJDQD1177-18|Polypedilum laetum
**Group[ 168 ] n: 2 ;**id: XJDQD1171-18|Polypedilum flavum XJDQD1172-18|Polypedilum flavum
**Group[ 169 ] n: 1 ;**id: XJDQD1178-18|Polypedilum nubeculosum
**Group[ 170 ] n: 4 ;**id: XJDQD1179-18|Polypedilum nubifer XJDQD1180-18|Polypedilum nubifer XJDQD1181-18|Polypedilum nubifer XJDQD1182-18|Polypedilum nubifer
**Group[ 171 ] n: 3 ;**id: XJDQD1183-18|Polypedilum sp. XJDQD1184-18|Polypedilum sp. XJDQD1185-18|Polypedilum sp.
**Group[ 172 ] n: 5 ;**id: XJDQD1186-18|Procladius crassinervis XJDQD1187-18|Procladius crassinervis XJDQD1188-18|Procladius crassinervis XJDQD1189-18|Procladius crassinervis XJDQD1190-18|Procladius crassinervis
**Group[ 173 ] n: 4 ;**id: XJDQD1191-18|Relictanum sp. XJDQD1192-18|Relictanum sp. XJDQD1193-18|Relictanum sp. XJDQD1194-18|Relictanum sp.
**Group[ 174 ] n: 1 ;**id: XJDQD1195-18|Rhamphomyia sp.
**Group[ 175 ] n: 1 ;**id: XJDQD1196-18|Rheotanytarsus erignus
**Group[ 176 ] n: 1 ;**id: XJDQD1197-18|Rheotanytarsus muscicola
**Group[ 177 ] n: 18 ;**id: XJDQD1198-18|Simulium sp. XJA XJDQD1199-18|Simulium sp. XJA XJDQD1200-18|Simulium sp. XJA XJDQD1201-18|Simulium sp. XJA XJDQD1202-18|Simulium sp. XJA XJDQD1203-18|Simulium sp. XJA XJDQD1204-18|Simulium sp. XJA XJDQD1205-18|Simulium sp. XJA XJDQD1206-18|Simulium sp. XJA XJDQD1207-18|Simulium sp. XJA XJDQD1208-18|Simulium sp. XJA XJDQD1209-18|Simulium sp. XJA XJDQD1210-18|Simulium sp. XJA XJDQD1211-18|Simulium sp. XJA XJDQD1212-18|Simulium sp. XJA XJDQD1213-18|Simulium sp. XJA XJDQD1214-18|Simulium sp. XJA XJDQD1215-18|Simulium sp. XJA
**Group[ 178 ] n: 19 ;**id: XJDQD1216-18|Simulium equinum XJDQD1217-18|Simulium equinum XJDQD1218-18|Simulium equinum XJDQD1219-18|Simulium equinum XJDQD1220-18|Simulium equinum XJDQD1221-18|Simulium equinum XJDQD1222-18|Simulium equinum XJDQD1223-18|Simulium equinum XJDQD1224-18|Simulium equinum XJDQD1225-18|Simulium equinum XJDQD1226-18|Simulium equinum XJDQD1227-18|Simulium equinum XJDQD1228-18|Simulium equinum XJDQD1229-18|Simulium equinum XJDQD1267-18|Simulium equinum XJDQD1268-18|Simulium equinum XJDQD1269-18|Simulium equinum XJDQD1270-18|Simulium equinum XJDQD1271-18|Simulium equinum
**Group[ 179 ] n: 2 ;**id: XJDQD1230-18|Simulium erythrocephalum XJDQD1231-18|Simulium erythrocephalum
**Group[ 180 ] n: 5 ;**id: XJDQD1232-18|Simulium sp. XJB XJDQD1233-18|Simulium sp. XJB XJDQD1234-18|Simulium sp. XJB XJDQD1235-18|Simulium sp. XJB XJDQD1236-18|Simulium sp. XJB
**Group[ 181 ] n: 1 ;**id: XJDQD1237-18|Stictochironomus sp.
**Group[ 182 ] n: 7 ;**id: XJDQD1238-18|Tabanus cordiger XJDQD1239-18|Tabanus cordiger XJDQD1240-18|Tabanus cordiger XJDQD1241-18|Tabanus cordiger XJDQD1242-18|Tabanus cordiger XJDQD1243-18|Tabanus cordiger XJDQD1244-18|Tabanus cordiger
**Group[ 183 ] n: 3 ;**id: XJDQD1245-18|Tanypus kraatzi XJDQD1246-18|Tanypus kraatzi XJDQD1247-18|Tanypus kraatzi
**Group[ 184 ] n: 1 ;**id: XJDQD1248-18|Tanytarsus sp. XJA
**Group[ 185 ] n: 2 ;**id: XJDQD1249-18|Tipula sp. XJB XJDQD1250-18|Tipula sp. XJB
**Group[ 186 ] n: 1 ;**id: XJDQD1255-18|Tipula sp. XJA
**Group[ 187 ] n: 1 ;**id: XJDQD1256-18|Tvetenia sp.
**Group[ 188 ] n: 1 ;**id: XJDQD1257-18|Wiedemannia simplex
**Group[ 189 ] n: 3 ;**id: XJDQD1264-18|Polypedilum sp. GY3 XJDQD1265-18|Polypedilum sp. GY3 XJDQD1266-18|Polypedilum sp. GY3


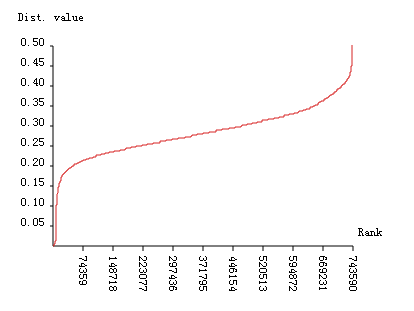


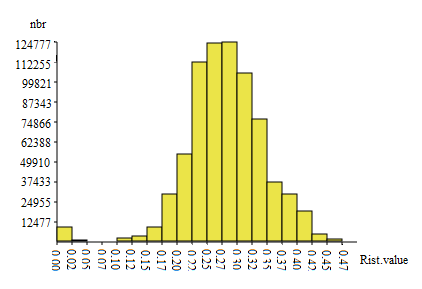


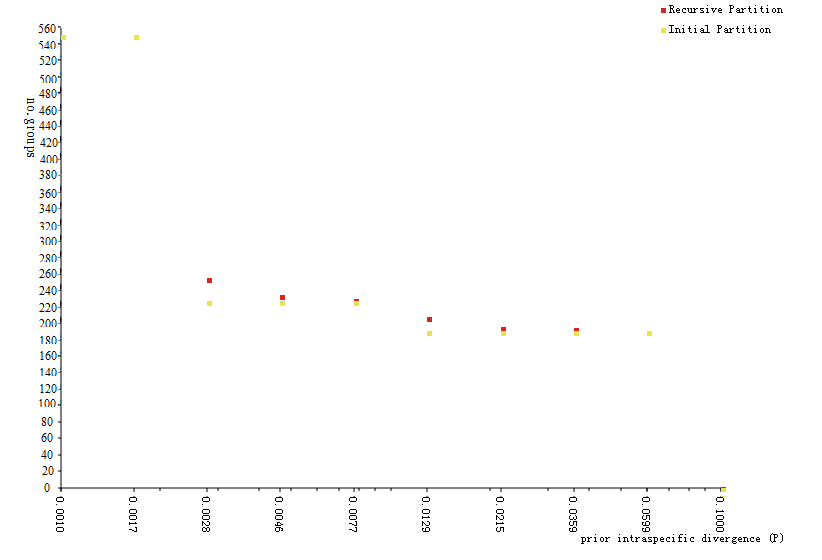


**Part III**

**Species delimitation** **annotated tree of the PTP and mPTP analysises for the combined dataset.**


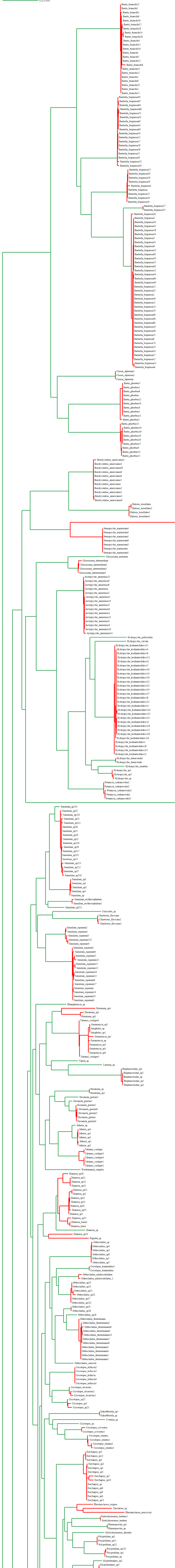


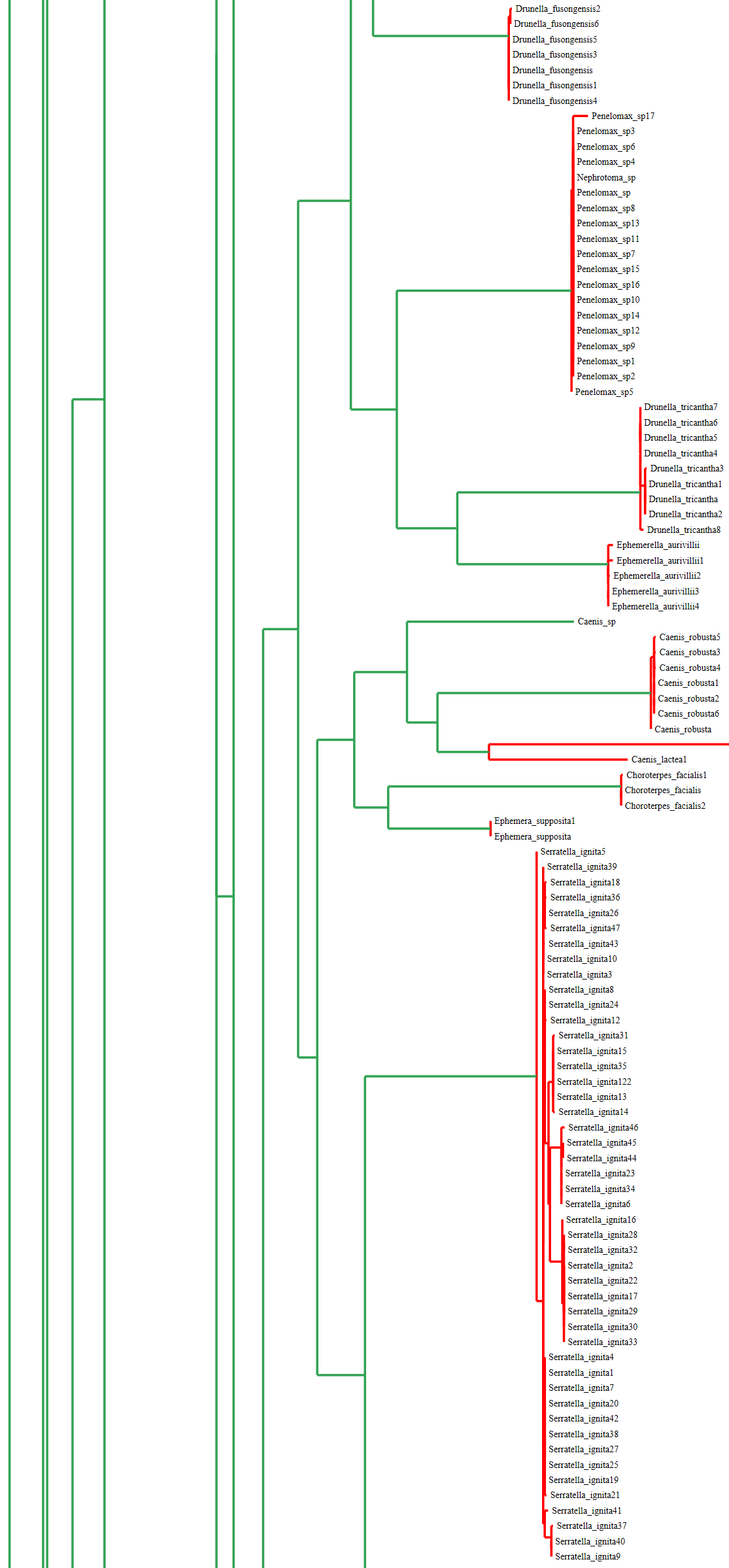


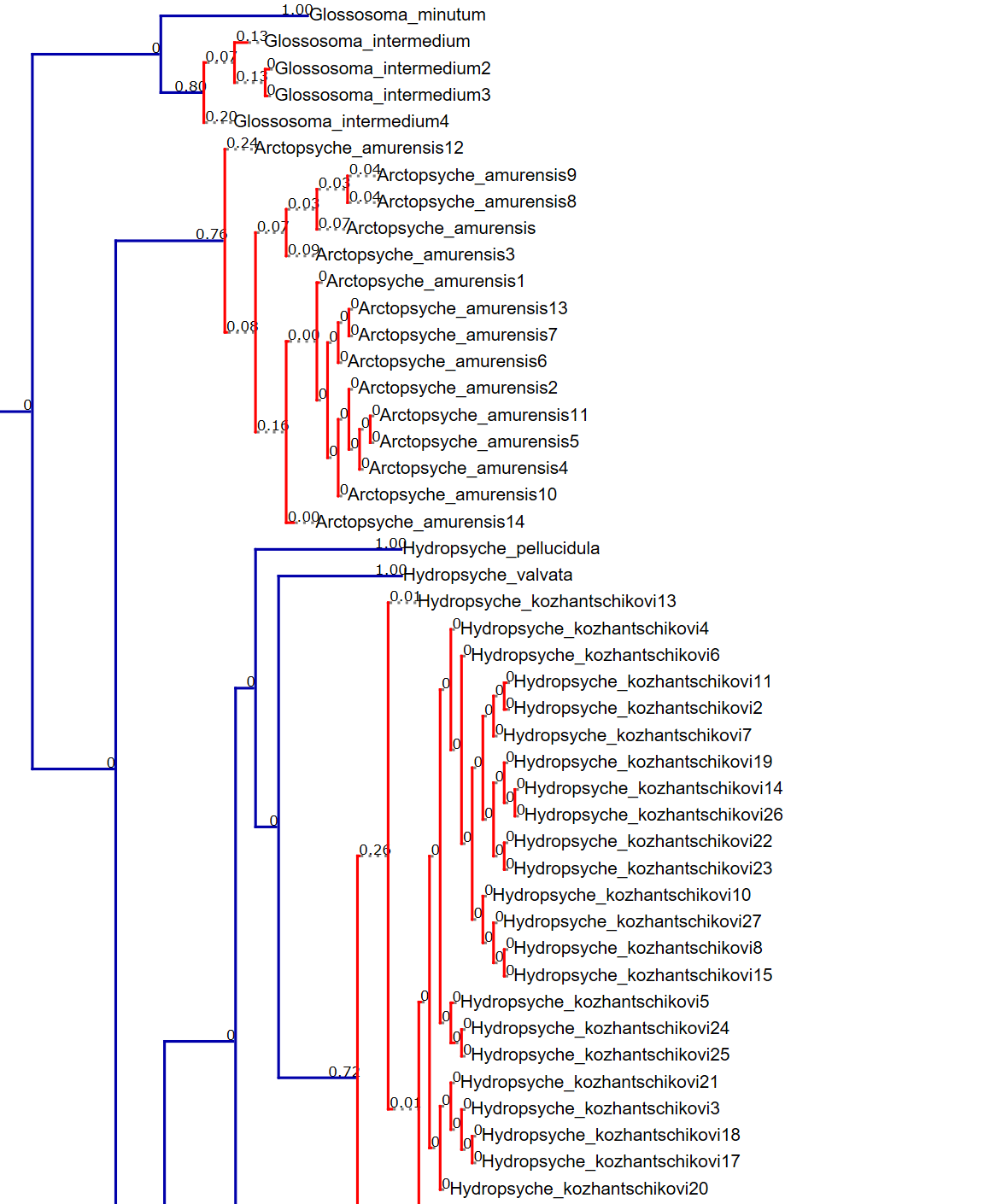

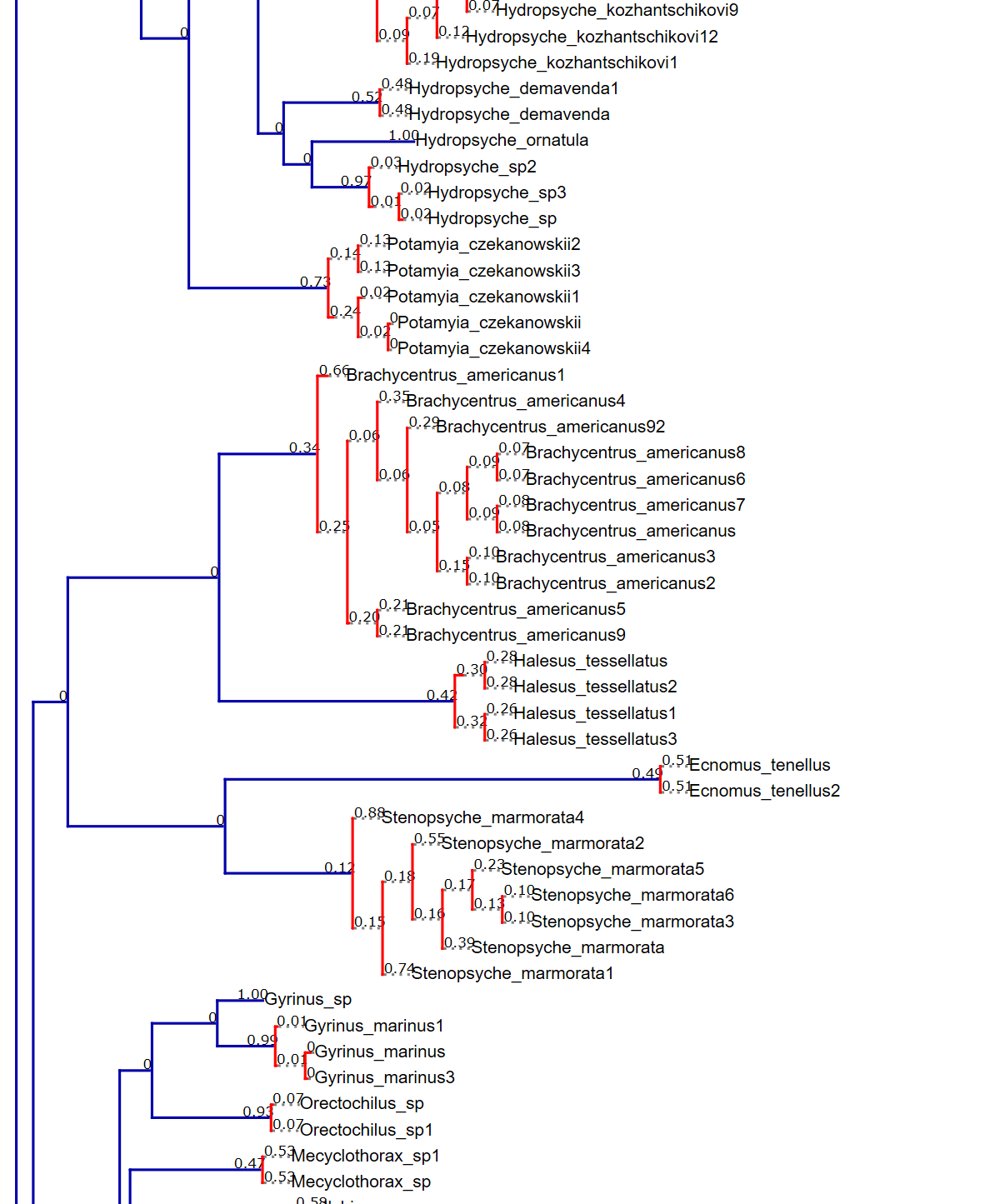

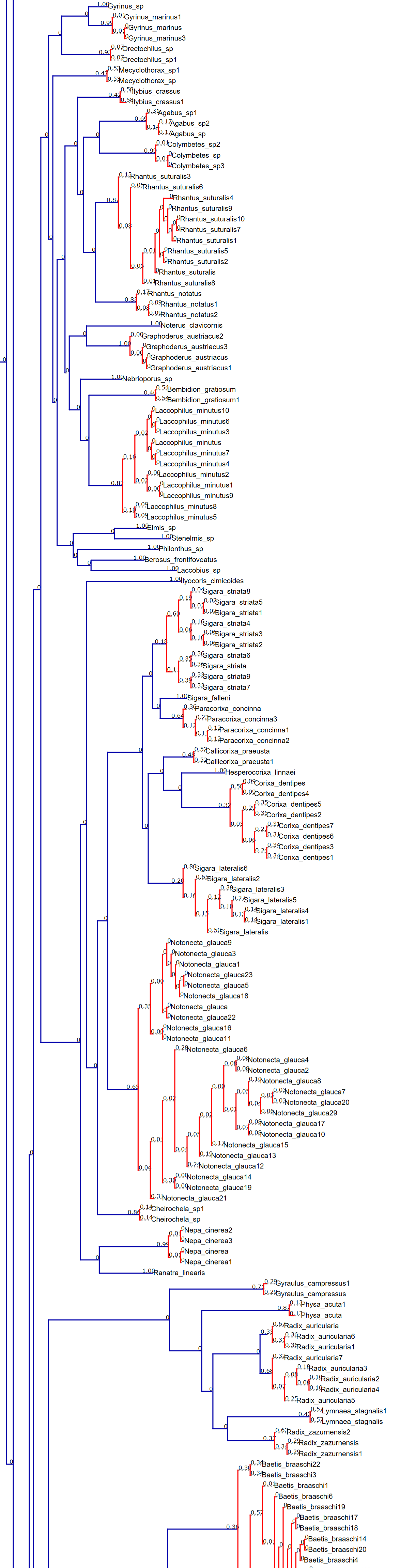


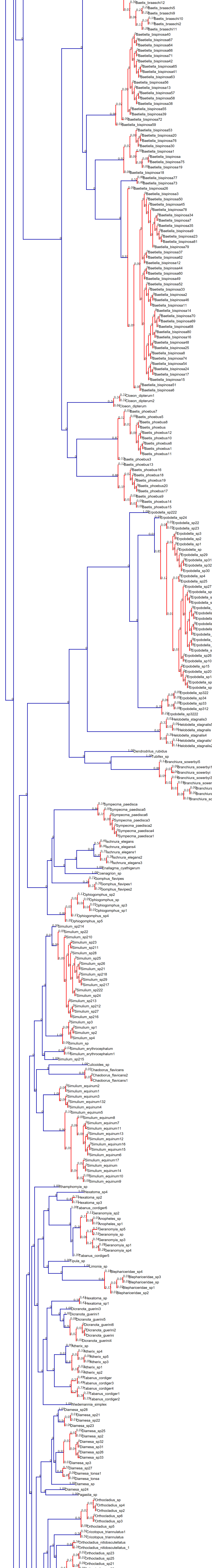


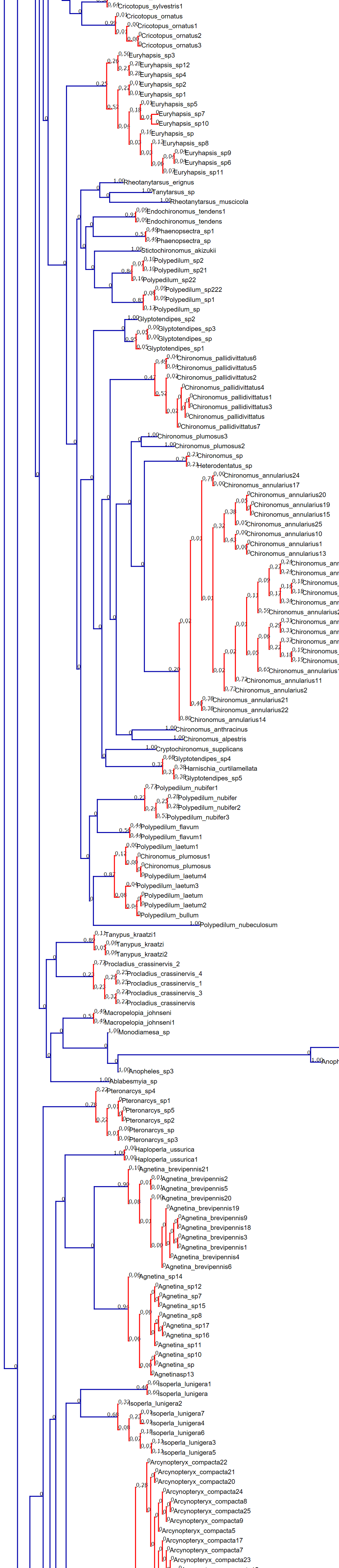


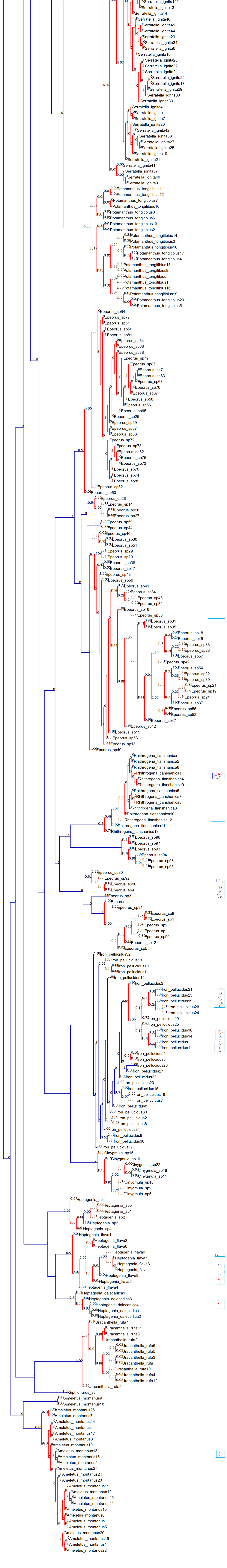

Supplement: Supplementary file 1 — Supplementary Material [file ECE3-11-5669-s001.docx]
